# ACcurate COnsensus Reporting Document (ACCORD) explanation and elaboration: Guidance and examples to support reporting consensus methods

**DOI:** 10.1371/journal.pmed.1004390

**Published:** 2024-05-06

**Authors:** Patricia Logullo, Esther J. van Zuuren, Christopher C. Winchester, David Tovey, William T. Gattrell, Amy Price, Niall Harrison, Keith Goldman, Alison Chisholm, Kirsty Walters, Paul Blazey

**Affiliations:** 1 Centre for Statistics in Medicine, University of Oxford, and EQUATOR Network UK Centre, Oxford, United Kingdom; 2 Leiden University Medical Centre, Leiden, The Netherlands; 3 Oxford PharmaGenesis, Oxford, United Kingdom; 4 Green Templeton College, University of Oxford, Oxford, United Kingdom; 5 Journal of Clinical Epidemiology, London, United Kingdom; 6 Bristol Myers Squibb, Uxbridge, United Kingdom; 7 Dartmouth Institute for Health Policy & Clinical Practice (TDI), Geisel School of Medicine, Dartmouth College, Hanover, NH, USA, previously at Stanford Anesthesia, Informatics and Media Lab, Stanford University School of Medicine, Stanford, California, United States of America; 8 OPEN Health Communications, Marlow, United Kingdom; 9 Global Medical Affairs, AbbVie, North Chicago, Illinois, United States of America; 10 Department of Medicine, University of British Columbia, Vancouver, Canada

## Abstract

**Background:**

When research evidence is limited, inconsistent, or absent, healthcare decisions and policies need to be based on consensus amongst interested stakeholders. In these processes, the knowledge, experience, and expertise of health professionals, researchers, policymakers, and the public are systematically collected and synthesised to reach agreed clinical recommendations and/or priorities. However, despite the influence of consensus exercises, the methods used to achieve agreement are often poorly reported. The ACCORD (ACcurate COnsensus Reporting Document) guideline was developed to help report any consensus methods used in biomedical research, regardless of the health field, techniques used, or application. This explanatory document facilitates the use of the ACCORD checklist.

**Methods and findings:**

This paper was built collaboratively based on classic and contemporary literature on consensus methods and publications reporting their use. For each ACCORD checklist item, this explanation and elaboration document unpacks the pieces of information that should be reported and provides a rationale on why it is essential to describe them in detail. Furthermore, this document offers a glossary of terms used in consensus exercises to clarify the meaning of common terms used across consensus methods, to promote uniformity, and to support understanding for consumers who read consensus statements, position statements, or clinical practice guidelines. The items are followed by examples of reporting items from the ACCORD guideline, in text, tables and figures.

**Conclusions:**

The ACCORD materials – including the reporting guideline and this explanation and elaboration document – can be used by anyone reporting a consensus exercise used in the context of health research. As a reporting guideline, ACCORD helps researchers to be transparent about the materials, resources (both human and financial), and procedures used in their investigations so readers can judge the trustworthiness and applicability of their results/recommendations.

## Abstract 2

Patricia Logullo and colleagues created an explanatory document that facilitates the use of the ACCORD checklist.

**Summary Points**

➢ The ACCORD (ACcurate COnsensus Reporting Document) guideline was developed to help report any consensus methods or techniques used in biomedical research.
➢ This document unpacks the elements included in the ACCORD checklist items and explains why and how to describe them.
➢ Examples for each ACCORD checklist item show it is possible and desirable to be transparent about the procedures used to reach consensus.
➢ A glossary of common terminology in consensus methodology is provided.
➢ This explanation and elaboration document is a thorough and practical guide on how to use the ACCORD checklist to report consensus research completely and transparently.

## Introduction: What is ACCORD?

The ACCORD (ACcurate COnsensus Reporting Document) guideline was developed to support rigorous reporting of consensus methods in biomedical research and clinical practice [1,2]. ACCORD is primarily designed to support the reporting of consensus exercises on topics related human health [3,4], however, the checklist may also be useful to researchers active in fields related to health, such as social and educational sciences.

ACCORD has been developed with the flexibility to apply to all methods or techniques used to achieve consensus, not only to the commonly used Delphi approach [1,3]. A non-comprehensive list of consensus methods used to measure, promote or gain consensus, and that can be reported using ACCORD is available in **Table 1** [5–14]. Researchers may choose to adapt a standard method, or to combine methods to fit their specific research needs. Other methods exist but all approaches may be reported using the ACCORD checklist. As consensus methodology will continue to evolve, the ACCORD checklist and this explanation and elaboration will require an update in due course to ensure they remain relevant to best-practice and new trends in the way consensus exercises are designed and conducted.

**Table 1:** Examples of some commonly used methods that can be applied, adapted, or combined to reach consensus.

**Consensus meetings**	In one or a series of meetings held in-person or online, a group of people discuss, in a structured and organised way, one or more topics with the aim of reaching consensus. The recommendations from the participants and the reasons for non-approval of items or topics are registered. The meeting structure is often highly individualised to the group and the topic under consideration.
**Consensus conference** [5]	A relatively rapid and inexpensive method of considering and evaluating aspects of new medical technologies when variation in practice exists. The first National Institutes of Health (NIH) conference was held in the United States in 1977 to help improve the translation of research findings into clinical practice. Prior to the conference, the available evidence on the topic is synthesised (by meta-analysis whenever applicable) for presentation at the conference by topic experts to a balanced and neutral panel comprising representatives from various aspects of professional and community life. After the presentations, the panel is asked to formulate their recommendations based on the scientific evidence presented. Formulating recommendations can be facilitated by asking the panel to indicate its support of specific recommendations from the literature using standard sentences as a grading scale for recommendations. It should be noted that are no gold standard process for decision-making or mechanisms for dissemination exist for consensus conferences; the exact methods used often vary.
RAND-UCLA Appropriateness* Method (RAM) [6]	RAND-UCLA appropriateness method (RAM) was created in the 1980s at the RAND corporation and the UCLA School of Medicine to help decisions in everyday clinical practice where trials are not available or do not provide evidence to support clinical decision-making. The rationale behind RAM is to determine ‘appropriateness’, defined as ‘the relative weight of the benefits and harms’ of a medical procedure. The process starts with a systematic review and synthesis of the literature available, the development of a list of indications and definitions which is provided to panellists. The ‘indications’ are hypothetical case scenarios designed to represent all the possible clinical variables a clinician may need to account for when recommending a particular procedure. A panel of typically between 7 and 15 members is then gathered and vetted extensively to ensure that panellists are the ‘best available’ to rate the appropriateness of the health procedure under consideration (can be a treatment; diagnostic test; prevention measure, etc). Appropriateness is indicated on a scale of 1 to 9, where 9 means the benefits outweigh harms. The first round is done individually (for example via mail or survey), and the second round is done in-person or via teleconference where summary measures are given from the first-round voting before discussion is allowed prior to a second and final vote (to allow members to change their position if they wish). Further rounds may be added to consider the ‘necessity’ of all approved medical procedures for a condition. *Appropriateness recommendations are often specific to the country in which they are developed.
**Delphi**	**Delphi**: Three characteristics define the Delphi consensus method: anonymity, iteration (over multiple rounds of voting), and controlled feedback. In a Delphi survey, a group of people (experts and non-experts) are consulted about one or more topics, statements, scenarios, quality indicators, core outcomes, etc. In the ‘traditional’ Delphi method, statements are generated by the panel members themselves as the first round is used as a ‘brainstorming’ round prior to any voting taking place [7]. Following the amalgamation of ideas presented Delphi panel members are then asked to vote anonymously (in the past, via post, most often now online), prior to receiving feedback in subsequent rounds on the group average agreement and how this compares to their own vote. Panellists are then given the opportunity to change their vote based on this controlled feedback [8]. **Modified Delphi**: A common modification to the traditional Delphi method is to present statements in round 1 that are based upon the results of a systematic or other type of evidence review. Other common modifications include: an in-person meeting to finalise Delphi recommendations; and changes to the 9-point Likert voting scale. When designing and reporting a modified Delphi, it is important to specify clearly in what way the Delphi approach was modified. **Real-time Delphi:** The real-time Delphi method is a round-less Delphi approach; participants are encouraged to re-visit the survey and re-rate items throughout the period in which the Delphi survey is live. Software is used to give feedback to participants on the web page in real-time — as opposed as waiting for the next round to receive summarised feedback. It removes the time taken in the standard Delphi approach for the survey administrator to evaluate the results and provide feedback to participants. Instead, participants see how other participants have answered the questions, and are able to modify their answers [9,10].
**Focus groups** [11]	A group of people is stimulated to discuss and share knowledge, ideas and experiences on a specific topic in an interactive environment. A moderator facilitates and encourages debate while ensuring that everyone has the space to express themselves and comment on each other’s views. Focus groups are widely used in qualitative research not aiming to reach agreement on a topic, but they can also be used with the objective of reaching consensus. They can be held in person or online, but when in person, a neutral space is preferred (not a place that represents just one side). One or more sessions, with the same or new participants, can be recruited until no new insights come up, and everyone has appreciated or learned each other’s views. Focus groups can be moderated to keep the discussions on the topic, to reach a consensus (for example, about how to solve a problem, or how to set priorities) or to generate ideas that will be submitted to other consensus exercises later.
**Nominal group technique - NGT** [12]	One or several face-to-face meetings (or online) are held and organised into iterative stages. The classic NGT involves four key stages: silent generation, round robin, clarification, and voting (ranking). In the first step, a group is silently asked to suggest ideas related to a topic or list of topics. A round-robin of panellists is then performed with each panellist sequentially being asked to feedback their idea until all new ideas have been exhausted. This process is repeated for all questions under consideration with participants having opportunity to clarify their ideas if they wish to do so. In the final stage, participants are asked to vote or rank the ideas put forward, usually using scales (like Likert). The group then discusses the aggregated summary of the voting or rating. The group is not anonymous and may include non-experts. It is recommended that groups consist of an uneven number of panellists (to achieve a majority) and that the number of panellists does not exceed nine to prevent dysfunctional group dynamics from affecting the process [12]. A trained facilitator makes sure every participant is given the opportunity to speak and vote. Facilitators can be non-experts on the topic of discussion but who hold credibility with the panellists (the Glaser method) or experts with subject-matter knowledge (the Delbecq method) [13,14].

Although designed to guide reporting, not conduct, ACCORD may alert researchers to problems and biases in consensus methodology that would otherwise be neglected. Still, the ACCORD guideline is not a methodological guideline; it neither specifies how researchers should undertake consensus research nor provides recommendations on the issues to deliberate when designing their agreement process.

### This explanation and elaboration document

This explanation and elaboration document unpacks the elements included in the ACCORD checklist items, explaining why they are important and how they can be addressed. **S1 Table** offers tips on how to report all the items even when journals impose word count limits. Together, the ACCORD reporting guideline statement [2] and this explanation and elaboration document support authors to report consensus exercises thoroughly and increase transparency of their methods.

Examples for each ACCORD checklist item show it is possible and desirable to be transparent about the procedures used to reach consensus. The examples cited in this document are shown solely to illustrate how a individual ACCORD checklist items can be reported. Examples have been sourced from the published, open access literature, most of them predating the publication of the ACCORD guideline, and all of them predating this document. They are therefore not idealised examples.

The systematic review conducted in the initial phase of the ACCORD checklist development [15] demonstrated pitfalls in the reporting of consensus methods within the current literature. Shortcomings in the reporting of consensus exercises means it is sometimes challenging to find ‘gold standard’ published examples. Consensus exercise manuscripts may lack the information necessary to satisfy the recommendations of the ACCORD checklist. This explanation and elaboration document therefore includes some ‘partially adherent’ reporting examples; those that adequately address some, but not all elements of the ACCORD checklist. Partially adherent reporting examples are acknowledged as such within the text. Inclusion of these examples allows us not only to highlight for readers the elements that are reported completely, but also those that are not, and to explain how these might be best addressed.

### ACCORD uses and users

The ACCORD materials – the reporting guideline and checklist [2] and this explanation and elaboration document – can be used by anyone drafting a report or manuscript (full or partial) about a consensus exercise used in health research. As a reporting guideline (see the Glossary in **Box 1** for “Reporting guideline”), ACCORD helps researchers to be transparent about the materials, resources (both human and financial) and procedures used in their investigations. The guidance also recommends that authors reflect on and discuss in the text the strengths and limitations, including possible biases involved in their decision-making processes [2].

The ACCORD guideline is not intended to provide a tool to assess the quality or rigour of published research [16]; rather it serves as a guide for the written reporting of consensus approaches. The checklist and this explanation and elaboration document may enhance readers’ understanding of the complexity and variety of consensus methods available and the factors that can influence the results of consensus exercises. We request authors address the reporting of certain items so that readers of their consensus exercise might judge the trustworthiness and applicability of their results/recommendations.

The terms ‘consensus’ and ‘expert consensus’ are heavily used in the medical literature, but not always in the context of reporting a consensus exercise that would be within the remit of ACCORD. For example, authors seeking to agree on the level of bias present in studies included in a systematic review, or assessing the ‘certainty of evidence’ using an approach such as Grading of Recommendations, Assessment, Development and Evaluation (GRADE) often use two evaluators and refer to a ‘consensus between researchers’. However, such processes are usually limited to two individuals (with an optional third to resolve conflicts) and are micro-steps undertaken as part of other study designs (for example systematic reviews or diagnostic accuracy studies). ACCORD is not designed to report ‘consensus’ carried out as part of another study design (for example, assessing risk of bias or piloting study materials).

### How to use this document

In this document, we explain the rationale for reporting the ACCORD checklist items, so that authors can understand the purpose of each individual item and its components. Some ACCORD checklist items may require authors to report multiple pieces of information, or elements (see Glossary for “Element”). In such instances, all elements should usually be reported, but there may be instances where the study design makes them unnecessary or not applicable.

To support the rationale behind reporting checklist items, examples of adequate reporting are provided to illustrate how authors may choose to satisfy the checklist recommendations. Authors are encouraged to adapt the phrasing to suit their context; there is no need to repeat the reporting style used in published consensus research. Items can be re-ordered if it makes sense to structure the manuscript differently, or if journal requirements request an alternative format.

To support readers of this document we have collated a set of commonly used terms related to consensus exercises. These have been placed in **Box 1** as a glossary of key terms used in the examples and explanations; terms included in the glossary are signposted within the text, for easy reference.

## Item Explanations and Examples

### Manuscript section: Title

#### T1. Identify the article as reporting a consensus exercise and state the consensus methods used in the title

*For example, Delphi or nominal group technique.*

Consistent with recommendations in other reporting guidelines [17–25], ACCORD suggests that authors reporting a consensus exercise should explicitly state the consensus methodology used in their title. In some cases, authors may employ mixed methods that include a consensus exercise. When mixed methods have been used, we encourage authors to consider other reporting guidelines that may be appropriate for their work (for example, AGREE-II [26] or COS-STAR [27]), and/or to consider whether the consensus exercise that is part of their project requires a separate publication (reported using ACCORD); this supports the greatest level of transparency. For example, the development of reporting guidelines may include a consensus exercise that forms just a component of the full methods used. Detailing the consensus methods used within the title of the published guideline might therefore be considered excessive, but would be appropriate in a separate publication focused on the consensus exercise.

Readers may opt to read only the title and the abstract of a study or may not have access to the full text. It is essential to let readers know that the conclusions or recommendations from the study were based on a group consensus. Also, it is important that librarians can correctly identify what type of study the paper is reporting [17,22] when indexing the manuscript, to avoid confusion with reviews, trials, and surveys from observational studies, for example. Another advantage of reporting the technique used in the title is to increase the chance that the paper is found by systematic reviewers or any other user [21,25].

The examples below show that it is possible and useful for the reader to report in the title the specific method(s) to achieve consensus — instead of only using the word ‘consensus’, which may also apply to genetics (consensus sequences) and machine learning or language techniques (where it can refer to agreement). By specifying the techniques used, the author lets the reader know if robust methods were in place to collect and present the participants’ views. In **Example 1**, the authors used the nominal group technique (NGT). The second (**Example 2**) is an example of the use of the RAND/UCLA appropriateness method (see **Table 1**). In **Example 3**, a single multidisciplinary consensus meeting was the singular approach to reach a consensus. In all these examples, the reader can immediately understand the context, the general objective of the study and the methods used to reach consensus.

Example 1.

“*Applicability of the interventions recommended for patients at risk or with delirium in medical and post-acute settings*: *a systematic review and a Nominal Group Technique study*.*”* [28]

Example 2.

“*Development and validation of Australian aphasia rehabilitation best practice statements using the RAND/UCLA appropriateness method*.*”* [29]

Example 3.

“*Genetic Aspects and Molecular Testing in Prostate Cancer*: *A Report from a Dutch Multidisciplinary Consensus Meeting*.*”* [30]

### Manuscript section: Introduction

#### I1. Explain why a consensus exercise was chosen over other approaches

Consensus methods are often chosen when evidence is absent or emerging, or when there is uncertainty in the existing research literature. Consensus recommendations may complement work done to identify the best available evidence. Consensus exercises for instance can be used to balance high certainty evidence with concerns such as: community preferences and priorities; resources; and/or equity considerations. Thus, consensus methods and evidence-based approaches are complementary and can be used together to improve the chances that recommendations are adopted by their target communities (additionally, see Glossary for “Clinical practice guideline”).

Whatever the reason for deciding to conduct a consensus exercise, authors should provide the rationale for seeking agreement, explaining whether evidence is currently absent, missing, or uncertain. If evidence already exists on a topic, authors may instead choose to explain why consensus is required to provide clarity. **Example 1** for instance, explains why consensus was required, and why the RAND/UCLA method was chosen. **Example 2** clarifies that, despite the presence of an existing consensus recommendation, there was a need to seek further clarity on the topic of contextual factors in healthcare. **Example 3**, establishes that there is an absence of evidence on the values or impact of patient involvement in research as a rationale to seek further guidance from the community.

Example 1.

“*For a heterogeneous topic such as surgical antimicrobial prophylaxis on which randomized controlled trials in pediatrics are lacking*, *the application of methods aiming to increase the homogeneity of clinical actions by neonatologists*, *infectious diseases specialists*, *pediatric surgeons*, *and anesthetists appeared useful and appropriate*. *For this reason*, *the RAND/UCLA approach was chosen instead of GRADE methodology*. *Through the RAND method*, *the participants discussed different clinical scenarios and elaborated statements based on the published literature and their clinical experience*.*”* [31]

Example 2.

“*The OMERACT’s [Outcome Measures in Rheumatology] broad definition is useful for understanding results in a clinical trial*, *in that it exists within a more historic paradigm that seeks to remove effects rather than enhance them*. *In this role it fails to resolve some of the confusion associated with the multitudes of ways contextual factors are presently defined (specifically*, *whether internal and external domains are potentially contextual factors)*. *For example*, *it does not include qualifiers to improve one’s understanding*, *and provides no guidance as to how clinicians may identify contextual factors within clinical encounters to enhance positive and minimize negative effects*. *Subsequently*, *the objective of this study was to create a consensus definition of contextual factors to better encapsulate this concept to both guide clinicians in clinical scenarios as well as broaden definitions for researchers*.*”* [32]

Example 3.

“*There is a plethora of literature illuminating the experiences of members of the public and professional researchers*, *and the process of PI [Patient Involvement] and its potential impacts*.^*6–9*^ *However*, *while studies report on use of PI in context of priority setting*,^*10*,*11*^ *the conduct of clinical trials*^*12*^ *and the identification of treatment outcomes*,^*13*^ *there has been relatively little examination of the values underpinning PI or about capturing and assessing the impact of PI effectively*.^*14–18*^ *Possible reasons cited for the limited number of studies evaluating the impact of PI are that evaluation is too difficult and that PI is of intrinsic value and as such needs no further justification*.^*16*,*19–21*^ *The authors of the present article acknowledge this latter perspective*. *However*, *we would argue that the current lack of a research evidence base around the impact of PI presents a challenge*, *since without such an evidence base it is difficult to ensure integrity of the PI endeavour*, *avoid potential adverse effects and ensure that it is adequately resourced*. *The modified Delphi study reported here was part of a larger Medical Research Council funded study (G0902155/93948) which aimed to review evidence on the values and impacts associated with PI*, *develop guidance on how these impacts can be assessed and contribute to the development of good practice standards for PI*.*”* [33]

#### I2. State the aim of the consensus exercise, including its intended audience and geographical scope (national, regional, global)

A clear aim helps the reader to understand the purpose of the consensus exercise. Specifying both the intended audience for the work (such as primary care physicians, researchers and/or policymakers) and the geographical scope (such as whether recommendations apply to international, national, or regional contexts and/or high-, middle and low-income countries) allows readers to understand where recommendations are intended to apply. Some consensus exercises may include participants at a regional or national level, but the work may have international applicability. If the geographical scope of the exercise is unclear, authors are encouraged to state that recommendations are based on the areas represented by those present in the panel (see Glossary for “Panel”).

The following **Example 1** succinctly specifies the three required elements – aim, intended audience and geographical scope – of the consensus exercise. **Example 2** reports the aim as a three-fold purpose that refers to the geographical scope. The intended audience for Example 2 is not stated in the example. We have therefore noted that Example 2 is incomplete as it implies the audience it aims to address, but to satisfy ACCORD recommendations fully authors would need to identify the target recipients of their work explicitly.

Example 1.

“*This research study aimed to develop the South African consensus on indicators for PC [palliative care] to assist clinicians to recognise a patient in need of PC*.*”* [34]

Example 2 (partially adherent — 2/3 elements).

“*Its purpose was threefold*: *it should (i) support EU/EEA countries in identifying meaningful indicators for PrEP [pre-exposure prophylaxis programs] programme monitoring*, *(ii) offer insight into the anticipated benefits and challenges of using specific data sources to report on these indicators and (iii) recommend a minimum set of core indicators to be collected and reported in a harmonised way across the EU/EEA*.*”* [35]

#### I3. If the consensus exercise is an update of an existing document, state why an update is needed, and provide the citation for the original document

A consensus recommendation is valid for the time, place and population for which it is generated. Therefore, consensus exercises, particularly those that look to provide assessment, diagnostic, and/or treatment recommendations may need to be repeated at set intervals. This helps to ensure that the outputs remain relevant in the context of newly published evidence, evolving clinical thinking and/or societal perspectives. When a consensus exercise is being undertaken to update previous work, referring to the previous work and the rationale for the update can help readers to understand not only why the update was conducted, but also how the information will add to existing knowledge. Including citation details for the previous publication allows readers to access the full details of the prior work and to see the evolution of consensus opinions over time.

**Example 1** states the consensus exercise is an update to existing recommendations, provides details of the scope of the update and also the authors’ rationale for updating the existing recommendations. What is missing from **Example 1**, however, is a citation to the previous guidelines. In contrast, **Example 2**, cites the guideline that preceded the current work. Similarly, **Example 3** reports and cites a published consensus statement and notes the perceived limitation of the previous work that motivated the reported update.

To meet the reporting criteria for item I3, we recommend authors:

➢ State if the work is an **update** to previously published work
➢ Report the **rationale for updating** the previous (existing) work
➢ **Include a citation** to the previous publication

Example 1.

“*This statement updates the previous OARSI [Osteoarthritis Research Society International] recommendations*, *incorporating literature published between January 2009 and March 2013*, *to scrutinize the safety and efficacy of new therapies for OA [osteoarthritis] and re-examine existing therapies in light of recent evidence*.*”* [36]

Example 2.

“*In 2013*, *the European Society for Primary Care Gastroenterology (ESPCG) published an evidence-based international guide for the use of probiotics in the management of specific lower gastrointestinal (GI) symptoms*.^*1*^ … *Since the publication of these statements*, *numerous relevant clinical studies of probiotics in the management of lower GI symptoms have been published*. *In the light of the new evidence available in this rapidly evolving field*, *the objectives of this publication are to update the systematic review and Delphi consensus*, *and to incorporate the new findings into the guidelines*.*”* [37]

Example 3.

“*The focus of the IHiPRN [International Hip Pain Research Network] specifically relates to ‘Hip-related pain in young to middle aged active adults’*. *There has been a consensus statement published previously that focused on FAI [femoroacetabular impingement] syndrome as a clinical entity and briefly discussed treatment options for FAI syndrome*.^*1*^ *While that consensus statement briefly recommended non-surgical treatments including rehabilitation*, *it did not specifically discuss evidence-informed recommendations for such treatments*. *To address this gap*, *the aim of this paper was to report the recommendations from the IHiPRN consensus meeting for physiotherapist-led treatments that improve pain and function in young to middle aged active adults experiencing hip-related pain*.*”* [38]

### Manuscript section: Methods

#### M1. If the study or study protocol was prospectively registered, state the registration platform and provide a link

**If the exercise was not registered, this should be stated.** *Recommended to include the date of registration.*

Prospective registration of the planned protocol for a consensus exercise is not a mandatory requirement for publication in a peer reviewed journal, but we recommend registration as best practice for increased transparency of the consensus exercise. Prospective registration allows readers of the published consensus findings to ascertain the extent to which the intended plan for the work was followed and what protocol amendments were made. Being aware of deviations from the intended approach gives readers the opportunity to consider the potential impact of these changes on the reported consensus findings.

In addition, public registration of intended research can help to avoid duplication and prevent research waste. There is no specific site, platform or repository for registering consensus exercises, but some options include registration on the Open Science Framework (https://osf.io) and/or publication of the protocol in a peer-reviewed journal. If the planned research was registered and/or the protocol published, reporting the name of the registration platform with the unique identifier assigned, and/or the publication citation details, assists the reader in sourcing the record/publication. If available, including a hyperlink to the registration record, registration platform or journal (as appropriate) further assists the reader. **Example 1** provides the Open Science Framework citation and identifies that this was published prior to commencing the study recruitment. **Example 2** indicates a pre-established study protocol with ethics approval and database registration reference. Many institutions require researchers to obtain ethical approval for any study involving humans or animals, such as surveys, and if required and obtained, it is optimal to report this (as well as any officially obtained waiver) along with other registrations, as the authors have done here.

Reporting the date of registration is helpful in communicating to the reader when the research was planned, which (particularly in rapidly evolving fields) may provide relevant context for the work, and insight into how long it has taken to complete.

To meet the reporting criteria for item M1, we recommend authors:

➢ Provide the **name of the registration platform** (database, journal, or similar)
➢ Provide the **registration record** details (such as a unique identifier), journal publication citation details, or similar, or hyperlink to the study protocol publication
➢ If not prospectively registered, provide a **justification** for that decision, including whether registration was considered and, if so, the reason that registration was not carried out

Example 1.

“*We preregistered a protocol before advertising the study (https*:*//osf*.*io/5h7bu)*. *Deviations from the preregistered protocol are outlined in Supplementary Material C*.*”* [39]

Example 2.

“*The study protocol was developed* a priori *and was approved by the UK Health Research Authority and by the Southwest Cornwall and Plymouth Research Ethics Committee (REC number 21/SW/0109)*. *The project was registered on the COMET [Core Outcome Measures in Effectiveness Trials] database*.^*11*^*”* [40]

#### M2. Describe the role(s) and areas of expertise or experience of those directing the consensus study

*For example, whether the project was led by a chair, co-chairs, or a steering committee and, if so, how they were chosen. List their names if appropriate, and whether there were any subgroups for individual steps in the process*.

Expertise and experience are linked concepts and are sometimes used interchangeably, but each provides distinct and useful information about an individual’s area(s) and depth of knowledge (see Glossary for “Expertise/expert”). Both aspects are helpful to report. Individuals leading the work may have areas of expertise that are relevant to the subject and objective of the consensus exercise, and/or they may have been involved in prior consensus approaches and have valuable experience of consensus methodology. Leading a consensus exercise does not necessarily require subject matter expertise/experience, but in instances where the people or person chairing the project has/have limited expertise/experience, they may convene a steering committee (see Glossary for “Steering committee”) of individuals to provide additional support and guidance to lead the project. For this reason, it is helpful to report the experience and expertise of those directing the consensus study and the role they take within the overall project: whether they are chairs, co-chairs, steering committee members, or similar. Reporting this information at the individual level, or task-specific level, allows readers to determine whether the individuals leading the consensus work had sufficient and appropriate knowledge to perform their role, or whether perceived knowledge gaps or bias may have affected the consensus findings. For the same reason, it is helpful to explain the role of the individuals involved in any subgroups that were convened to deliver parts of the project, such as to conduct a systematic review (see Glossary for “Project committee” and “Executive committee”).

The example names all the individuals who were involved in leadership positions at the different stages of the consensus activity as well as the different subgroups that were convened, and by whom, to lead specific aspects of the work. The authors also describe the combined expertise in each subgroup and its relevance to the assigned task.

To meet the reporting criteria for item M2, we recommend authors:

➢ Provide details on **who** led the consensus exercise and their roles, such as any chairperson(s), steering committee and subgroups convened to perform specific tasks
➢ Detail the **experience and expertise** of the individuals who were appointed to each leadership position
➢ Explain the **method of appointing** to leadership positions, such as self-appointment, personal invitations from the chair, or formal selection through open competition.
➢ Include the **names and roles** of the individuals in each leadership position so that that it is clear where individuals had more than one role

Example.

“*Study groups and participants DM*, *TN*, *DMN*, *and PRW oversaw the PC-COS [Post Covid-Core Outcome Set] project*, *identified and invited individuals with relevant expertise to form the core author group*, *and were responsible for the study methods and day-to-day project management*. *The core author group had expertise in methodology*, *various fields of clinical medicine and clinical research*, *psychology*, *epidemiology*, *public and global health*, *and public and patient engagement*. *A methods group (DM*, *TN*, *WDG*, *JP*, *NSc*, *NSe*, *FS*, *AT*, *DMN*, *and PRW) was established to develop and oversee the project methods*. *A PC-COS steering committee was established by DM*, *TN*, *DMN*, *and PRW in collaboration with the WHO [World Health Organization] Clinical Characterisation and Management Team (WDG*, *JVD*, *JP*, *and NSc); participants were identified and invited through expert networks including ISARIC [International Severe Acute Respiratory and emerging Infection Consortium]*, *WHO*, *and the COMET [Core Outcome Measures in Effectiveness Trials] Initiative*, *and support groups for people with lived experience*. *The PC-COS steering committee comprised 46 members from 13 countries*, *including health-care professionals*, *researchers representing a range of medical fields*, *methodologists*, *WHO representatives*, *and people with post-COVID-19 condition and their carers*, *and was actively involved in the design and conduct of this project (see appendix 1*, *pp 1–5*, *for further details of the PC-COS project steering committee members)*.*”* [40]

#### M3. Explain the criteria for panellist inclusion and the rationale for panellist numbers

**State who was responsible for panellist selection**.

The M3 ACCORD checklist item combines three important elements: the criteria for inclusion of panel members, an explanation on how the number of panellists was reached and who was responsible for selecting panel members (see Glossary for “Panel”). Choosing the number of panellists can be challenging for consensus exercises, as there is no ‘sample size calculation’ and no gold standard for measuring panel diversity (see Glossary for “Sample size”). The number of panellists is often justified by referring to previous consensus exercises on similar topics or based on diversity. Recommendations for panel sizes vary and are frequently based on feasibility (‘as many as we can get’; ‘as many as we can analyse’) and rule-of-thumb numbers, rather than statistics.

As the number of panellists will vary depending on topic, aim, resources available, and method of consensus measurement/generation, authors should report the reason behind their chosen number, even if it was reached by convenience or ‘snowballing’ (see Glossary for “Snowball sampling”). As you will note from looking at the examples, providing a comprehensive explanation of all three elements in M3 can be tricky. We were unable to locate an example that satisfied all three.

**Example 1** covers two of the three elements: who made the decisions and how panellists were chosen. The example explains that the co-chairs selected the panel based on publication records and countries represented; the reader will be able to judge whether the results are truly international. However, **Example 1** misses the rationale for the chosen panel size. It is implicit that, by including a representative from “at least 100 countries”, they were aiming to represent a diverse population and encompass a broad range of ideas, but it would have been helpful to state this explicitly in the explanation.

Although **Example 2** explains a lot about the process of recruitment, it is marked as partially adherent because it also reports only two of the three elements explicitly: how the panellists were chosen and how the (minimum) number of panellists was decided. Who made the decisions to include panellists is not reported, leaving the reader to infer from the context that it was likely to have been the authors. **Example 3** states that the steering committee selected the panellists and explains that the number of panellists chosen was based on the snowballing rationale of ‘as much as we can get in a period’. However, although the example outlines key considerations involved in panellist selection – geographical and disciplinary diversity – the authors do not provide an explicit set of parameters or criteria against which each panellist was assessed. For this reason, the example is also marked as partially adherent.

According to the M3 checklist item of ACCORD, authors must reveal:

➢ **Who** made decisions on the panel composition
➢ **How they chose** panellists (criteria used)
➢ **How they decided on the number** of panellists targeted

Example 1 (partially adherent — 2/3 elements).

“*The four co-chairs (J*.*V*.*L*., *A*.*B*., *A*.*K*. *and A*.*E*.*-M*.*) identified a core group of 40 academic*, *health*, *NGO*, *government and policy experts from 25 countries and territories*. *Selection by the co-chairs was primarily based on publication record and engagement on COVID-19 issues as well as online biographies*. *Twenty-nine of these experts were well known to the chairs while seven were suggested through snowball sampling to result in geographical and gender equity among the core group of 40* … *In proposing experts*, *co-chairs focused on identifying at least one representative from at least 100 countries*. *One co-chair (J*.*V*.*L*.*) took responsibility for reviewing the suggestions*, *with support from a research assistant who shared recent publications and a professional biography for every proposed co-author*. *Many initial suggestions were of leading experts with whom the co-chairs had previously collaborated*.*”* [41]

Example 2 (partially adherent — 2/3 elements).

“*In Delphi exercises*, *a minimum of 12 respondents is generally considered to be sufficient to enable consensus to be achieved*, *larger sample sizes can provide diminishing returns regarding the validity of the findings*.^*34–39*^ *Nevertheless*, *Delphi sample sizes depend more on group dynamics in reaching consensus than their statistical power*.^*40*,*41*^ *A non-probability purposive sample of ninety-six participants were invited via email to participate in this Delphi survey. Sampling was purposive to ensure that invited participants met the inclusion criteria. All participants were required to be 18 years or above, fluent English speakers, actively conducting research in obesity or obesity-related fields, and affiliated with an academic institution from an OECD country. The invited participants were either members of the Obesity Network (n = 34), academics known to members of the Obesity Network (n = 45), or authors of published articles relating to obesity and big data identified from the first paper in this series [5] (n = 17). To complete the Delphi process, participants were required to respond across all three rounds. Therefore, those who did not respond to Round 2 were not invited to participate in Round 3. A dropout rate of 20% was expected over the three rounds, in accordance with previous Delphi studies.*^*32*,*42*^ *This study aimed to recruit and complete the process with 30 experts*.*”* [42]

Example 3 (partially adherent — 2/3 elements).

“*Delphi participants were identified by the steering committee*, *from authors of relevant publications via a call to participate on social media (e*.*g*., *Twitter)*, *and through personal recommendations*, *including experts recommended by other Delphi participants*. *The steering group identified participants to achieve geographical and disciplinary diversity and include key stakeholder groups*, *e*.*g*., *researchers (statisticians/data scientists*, *epidemiologists*, *machine learners*, *clinicians*, *radiologists*, *and ethicists)*, *healthcare professionals*, *journal editors*, *funders*, *policymakers*, *healthcare regulators*, *patients*, *and the general public as end users of prediction models from a range of settings (e*.*g*., *universities*, *hospitals*, *primary care*, *biomedical journals*, *non-profit organisations and for-profit organisations)*. *No minimum sample size was placed on the number of Delphi participants*. *A steering group member checked the expertise or experience of each identified person*.*”* [43]

#### M4. Describe the recruitment process (how panellists were invited to participate)

*Include communication/advertisement method(s) and locations, numbers of invitations sent, and whether there was centralized oversight of invitations or if panellists were asked/allowed to suggest other members of the panel.*

Both M3 and M4 ACCORD items are about the characteristics of the panel members involved in the consensus process (see Glossary for “Panel”). However, while M3 is focused on the criteria for the selection and size, M4 deals with the **process of inviting** people to participate (See Glossary for “Recruitment”). Again, three elements must be reported: the communication method used to send the invitations, the number of invitations sent and whether the recruitment was centralised (controlled by a steering committee) or whether panellist were recruited through snowballing (see Glossary for “Snowball sampling”) or open invitation. Authors may use more than one method to contact potential participants, accounting for equally accessible options (for example when attempting to recruit different age-groups). (See Glossary for “Accessibility”.) Reports should clearly detail all the methods applied and the intended target groups in each case.

In **Example 1**, the number of invitations sent is irrelevant, as recruitment was open, meaning that the number of invitations to be sent was unrestricted, but the use of posters did target specific groups of potential panellists. In **Example 2**, combining both purposive and snowballing recruitment, the initial number was given, and the authors explain how they contacted the potential participants. It also shows measures taken to ensure that those initially contacted were able and supported to participate, reducing the potential dropout of panellists (see Glossary for “Drop-out”). Reporting these details allows readers to understand how the efforts in recruiting might have resulted in fewer or more participants. For other researchers trying to reproduce the method, it shows the resources required.

We have included a partially adherent example for illustrative purposes. **Example 3** reports that e-mails were used, and implies there was a closed list, but it does not clarify how many invitations were sent, nor if panellists were able to invite others. Studies that aim to get ‘the right panellists’ instead of focussing on a large group often use ‘closed’ approaches. This may apply to specialised or niche areas where authors focus on inviting panellists only with a specific set of expert characteristics (see item M3).

The recruitment process is the focus of M4; authors should report:

➢ The **communication methods** used to invite people
➢ The **numbers** or **types of invitations** actually posted
➢ Whether the invitees could **recruit more** people (snowballing) or whether the recruitment was **restricted** to the invited panellists

Example 1.

“*2*.*3*. *Participants and recruitment*.

*The recruitment took place between June and October 2021. Eligible criteria for participation included women who were aged between 50 and 70, had been offered breast screening, had the capacity to give consent and resided in Newport West, Wales*.

*A recruitment poster was circulated via emails and social media (e*.*g*., *Facebook and Twitter) through community networks and the eight GP practices in the areas*. *Hard copies of the poster were on display in GP practices*, *local libraries and community centres in the area*. *GPs were not involved in the recruitment process*. *Participants contacted the researchers directly if they were interested to take part*.*”* [44]

Example 2.

“*A purposive sampling strategy was used to ensure that the participants were committed members of the hospital at different stages*. *Clinical nurse specialists in each hospital provided mailing lists of potential participants*, *who were invited to participate via an e-mail providing general information on the study’s nature and purpose*. *Additional nurses were recruited via direct contact during an online meeting and by snowballing*. *In total*, *17 nurses agreed to share their experiences*. … *The principal investigator set a date for the NGT sessions and then invited the potential participants via phone or e-mail*, *which included notification of the inclusion criteria for participation*. *Only those who fit the criteria were recruited*. *They were given two weeks to make the decision to participate and sign the consent form*. *Until they completed the consent forms*, *they were not divided into groups for the sessions*.*”* [45]

Example 3 (partially adherent — 2/3 elements).

“*An initial list of individuals was identified through searches of electronic databases (e*.*g*., *Cochrane Library [including the HTA database]*, *Scopus) and contacting key organizations undertaking rapid reviews (e*.*g*., *Cochrane Rapid Reviews Group and Health Technology Assessment international)*. *We aimed to include authors from as many countries as possible*. *Email addresses were collected from personal contact lists and publicly available sources (e*.*g*., *organizational websites)*. *All emails were personalized to individuals and all contacts were assured confidentiality of their responses*, *with the aim of encouraging participation and openness*.*”* [46]

#### M5. Describe the role of any members of the public, patients, or carers in the different steps of the study

The incorporation of members of the public, patients and caregivers is relevant for studies in health [47] (see Glossary for “Experience”). Patient and public involvement (frequently referred to as ‘PPI’), co-production or patient engagement are terms used by some journals that require authors to report the PPI input in research or in writing manuscripts and plain language summaries. Patients are also increasingly participating in consensus panels and in the design or steering committees of consensus exercises (See Glossary for “Steering committee”).

ACCORD requests authors to report on the participation of patients and the public in projects using consensus but not just to ‘tick a box’: ACCORD requires a full description. It is important to specify how patients and the public were involved, and at what stages of the consensus exercise. It is equally important to state barriers to PPI as this helps make the research replicable and to identify barriers for later problem solving. Initiatives that involved clinicians only, for example, should simply declare so and explain why.

In some situations or steps of the consensus process, it may be not relevant to include patients, and authors should report it. However, as in **Example 1** shown below, authors should explicitly report which steps patients participated in. In **Example 2**, the authors explain in detail the roles played by people living with the health condition (an anterior cruciate ligament tear). This shows researchers involved patients, but only those with a specific experience — just as sometimes a certain level of expertise is expected from panellists (see Glossary for “Expertise/expert”). **Example 3** reports the stages where patients participated and how, as did **Example 4**, where the patient representative was a non-voting but important contributor.

ACCORD recommends that researchers report:

➢ **Whether** they included PPI or not
➢ The **roles** of the public or patient representatives in each step of the study or consensus exercise: this includes **what** they did, **how**, and **when**

Example 1.

“*Public panel - Members of the public*, *as key stakeholders*, *were invited to participate in Round 1 of the Delphi exercise*. *A simplified version of the Round 1 questionnaire presented to the expert panel was adapted for a non-expert audience (see Supplementary Note 1)*. *There were no qualifying criteria or prior knowledge required for participation*. *The public panel were recruited through the VOICE platform (https://www.voice-global.org/)*, *an organisation which comprises of members of the public across the world who volunteer to contribute their insights to health research*.*”* [48]

Example 2.

“*One individual with lived experience of ACL tear (and ACL reconstruction (ACLR)) and four clinicians (ie*, *physiotherapists and orthopaedic surgeons) contributed to the priority theme setting for the OPTIKNEE consensus*. *One patient partner and one clinician (sports and exercise medicine physician) were authors on the risk factor review*,^*29*^ *and one additional patient and clinician partner provided feedback on one of the intervention reviews*.^*30*^ *A patient partner and a clinician (physiotherapist) provided feedback on this manuscript*.*”* [49]

Example 3.

“*Patient partners were collaboratively involved at key stages of the study*. *Three patient partners were recruited to the research team and were involved in refining the focus of the research questions*, *in development of the search strategy and interpretation of results of the systematic review*, *in discussions identifying the need for development of guidelines*, *and in selecting the items for the original GRIPP checklist*. *Furthermore*, *the patient partners assisted in developing the electronic survey for the first phase of the Delphi survey consensus process and were instrumental in assisting in recruitment to the Delphi study and in collation of comments from each Delphi survey round and contributed to adapting items for GRIPP2*. *The consensus meeting involved eight patient partners in total*, *and the three patient partners recruited to the research team were involved in the write-up of the study and are coauthors in papers*. *More detailed information of their contribution to the development of GRIPP is described using GRIPP2-SF in table 3⇓ and used to populate the BMJ PPI guidance in box*.*”* [50]

Example 4.

“*Patient and public involvement statement*. *A leadership representative from the Fabry International Network (FIN)*, *JJ*, *was invited to participate in the project in a non-voting role*. *The representative reviewed and approved the initial protocol and round 1 questionnaire and facilitated the involvement of three patients with FD (one from the USA and two from outside the USA) in reviewing these materials*. *This ensured that any appropriate feedback from the patients could be incorporated into materials before distributing the round 1 questionnaire*. *Additional roles of the FIN representative included capturing these patients’ views on the outcomes of the initiative and reviewing and approving the final study report*.*”* [51]

#### M6. Describe how information was obtained prior to generating items or other materials used during the consensus exercise

*This might include a literature review, interviews, surveys, or another process*.

Individuals participating in consensus exercises may receive briefing or reference materials about the topic and/or about their role, and an initial set of items or statements to vote on, rate or rank the order (see Glossary for “Ranking” and “Rating”). It is important for readers to know where and how this information was obtained and how these initial materials were created. Information could be obtained from a single source or from multiple sources, such as documented reports of personal experience, interviews or workshop outputs, survey responses, or bibliographic searches (systematic or non-systematic). Relevant information sources will vary for different consensus exercises and topics, and the area of biomedicine under consideration.

Regardless of the consensus topic and the how information has been summarised, it is important for authors to report fully the origin of the information used. Reporting the sources used, and the rigour with which information is obtained, adds context and helps readers to assess whether this was a strength or a limitation of the process. It is also possible that if authors select a limited set of sources from which to draw and summarise information or to generate their initial statements it could bias the result of the consensus exercise. Source selection and its subsequent recording are therefore an important part of transparent consensus reporting.

In the examples that follow, **Example 1** explains that the initial information for the consensus exercise was gathered via a survey and details the survey platform that was used. **Example 2** highlights how data and themes explored in the consensus exercise were identified (in workshops) and includes information about the publication that was discussed during the sessions and how many workshops were organised. It also reports the number and profession of participants. **Example 3** reports a range of dates over which qualitative data were captured, the methods by which these data were analysed and how they were combined with literature-based data to inform the development of a Delphi survey. Finally, **Example 4** states that the consensus exercise began with a time-limited idea-generation activity, reports the dates of that activity and elaborates on the process of collecting and merging the information.

To describe fully how information informed the generation of statements, evidence summaries, or other materials we recommend authors:

➢ Provide the **type of information source(s)** used (such as evidence synthesis, interview(s), or survey)
➢ **Explain how information** gathered from interviews, surveys or reviews was **used** in the consensus process (for example, to generate items for participants to vote on).

Example 1.

“*An initial online survey (SurveyMonkey; Momentive*, *San Mateo*, *California*, *USA) was used to invite patients*, *carers*, *healthcare professionals and members of the public to suggest evidence of uncertainties connected with sustainable perioperative care*. *Respondents were asked to state*, *via free-text boxes*, *what questions they felt needed to be answered by future research to help make perioperative practice more environmentally sustainable*. *To help respondents to consider the full scope of the perioperative patient journey*, *we asked them to consider the preoperative*, *intraoperative and postoperative phases and also invited any further suggestions*.*”* [52]

Example 2.

“*Three expert workshops (participant total n=42)*, *including members of the public*, *academic and user-researchers*, *researcher/clinicians*, *research funders and research managers were conducted to generate qualitative data*. *The range of normative*, *substantive and process-related values underpinning [public involvement]*, *identified in a previously conducted literature review*,^*22*^ *were discussed*, *to identify concepts to be explored at round 1 (R1) and round 2 (R2) of the subsequent modified Delphi survey”*. [33]

Example 3.

“*A published systematic literature review (SLR)*^*9*^ *identified all the outcomes documented in studies since 1990 involving patients who had undergone surgery for CES [cauda equina syndrome]*. *The outcomes from the SLR were combined with the outcomes identified from the qualitative interviews to form those initially rated on within the Delphi Survey*. *These qualitative interviews had been conducted by NS with 22 patients treated at The Walton Centre between 2007 and 2016 for CES*. *A sampling frame was applied to ensure patients with a range of CES severities (CESI or CESR) and different times since the operation were interviewed*. *Semi-structured interviews were conducted with a topic guide (S1 File) and involved patients’ describing their experience of CES in a chronological manner to ascertain the relevant outcomes and the lived experience of the condition*. *Interviews were audio recorded*, *transcribed and with the assistance of NVivo (version 10)*, *were coded using an inductive approach to identify outcomes*. *NS led the analysis process and was supported by AN*.*”* [53]

Example 4.

“*2*.*4*.*1*. *Activity 1*: *Brainstorming (June–July 2021)*

*During the brainstorming activity (also known as ‘item generation’), participants generated as many statements (items of support) as they thought appropriate in response to the focus prompt: ‘Something that would help me to go for breast screening is[…]’. The activity was open for 5 weeks. For online participation, an invitation was emailed to participants, which contained the link to the study site, a unique login code, and instructions on how to take part. For offline participation, a sheet that contained the focus prompt and instructions on participation was provided*.

*The Key Words in Context method was used to review the raw statements generated by participants*.^*34*^ *The process involved reviewing the raw list*, *removing duplicates*, *splitting compound statements and checking for grammar and spelling mistakes*. *For example*, *the statement ‘Not mistreated or judged due to my background or language barrier’ was separated into two statements ‘Not feeling mistreated or judged due to my background’ (statement 4) and ‘Not feeling mistreated or judged due to my language barrier’ (statement 5)*.*”* [44]

#### M7. Describe any systematic literature search in detail, including the search strategy and dates of search or the citation if published already

*Provide the details suggested by the reporting guideline PRISMA and the related PRISMA-Search extension*.

A ‘search strategy’ is more than ‘the main key words used’ in a literature search. Each database is different and so the search strategy for each is slightly different. It includes not only the search terms, but the database version and platform information, the Boolean operators, the database fields that each term was searched in, the database-specific syntax, any search filters or limits used and the date that the search was run. The exact search, exactly as run in each of the databases, should be provided. This information is essential for understanding exactly what was searched for and how this was done and allows the search to be replicated.

The PRISMA-Search reporting guideline was published in 2021 and includes a 16-item reporting checklist specific to systematic literature searches, covering the information sources and methods, search strategies, peer review and record management steps involved in conducting a systematic search [54]. The PRISMA-Search checklist is available open access and should be used to guide robust and complete reporting of the details of any literature review conducted as part of the consensus exercise. The search strategy can be included in the supplementary materials/publication appendices, or the review may be disseminated as a separate publication and cited in the consensus publication.

**Fig 1:**
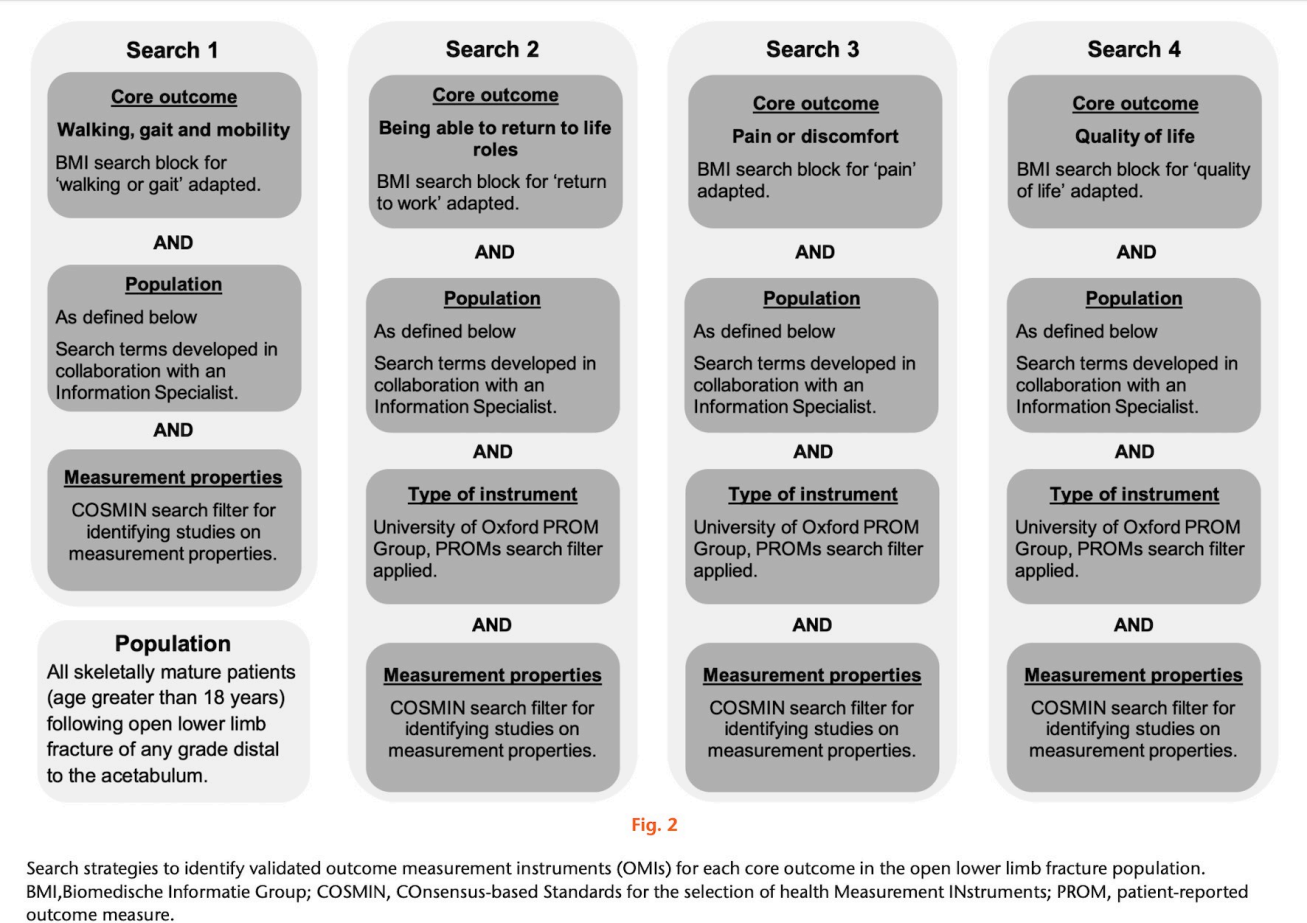
Figure by Aquilina et al. *Bone Joint Res*. 2023;12:352–361 (DOI: 10.1302/2046-3758.126.BJR-2022-0280.R1). This work is licensed under a CC-BY-NC-ND 4.0 licence and with the permission of the authors.[56].

The three following examples describe the bibliographic databases or the grey literature interrogated by the systematic searches used. All examples give the dates when searches were conducted. **Example 1** gives the citation for the published search strings. **Example 2** provides how references were managed and signposts the full search strategies in the appendix file. Information about systematic reviews can also be presented graphically; **Example 3** shows how searches can be linked in figures (see **Fig 1**) and supplementary materials.

To meet the reporting criteria for item M7, we recommend that authors provide the following details:

➢ **Platform**(s) covered, including grey literature if relevant
➢ Whole **search strategy** (with terms, filters and connectors) used
➢ **Date range** of search
➢ **Date that each search** was conducted

Example 1.

“*An extensive list of potential post-COVID-19 condition outcomes*, *to inform the COS [core outcome set] consensus process*, *was created using data from a living systematic review*,^*2*^ *clinical trial protocols*, *and additional studies*, *including a survey led by people with lived experience of post-COVID-19 condition*^*12*^ *and references suggested by steering committee members*. *For the living systematic review*, *MEDLINE*, *CINAHL (EBSCO)*, *Global Health (Ovid)*, *the WHO Global Research Database on COVID-19*, *and LitCovid were searched for articles published in English from Jan 1*, *2020*, *to March 17*, *2021; further details of the search strategy used for the living systematic review*, *including search terms*, *are presented elsewhere*.^*2*^ *As part of the living systematic review*, *an additional search of Google Scholar was done on March 17*, *2021*, *and the first 500 titles were screened*.^*2*^ *We manually reviewed selected studies published after the systematic review search period*, *as well as other systematic reviews*, *narrative reviews*, *opinion papers*, *and relevant references cited in the identified articles*. *Clinical trial protocol data were extracted from ClinicalTrials*.*gov and the International Clinical Trials Registry Platform*.*”* [40]

Example 2.

“*Systematic searches for randomized controlled trials and clinical*, *controlled trials were undertaken using the following databases on 15 May 2020 limiting the time to 2016 – 15 May 2020*: *- Ovid MEDLINEI ALL 1946 to May 14*, *2020; - Embase Classic+Embase 1947 to 2020 May 14; - Cochrane Central Register of Controlled Trials (CENTRAL)*. *All search strategies can be found in Appendix 1*: *Search Strategies*. *We did not search trials registries*, *grey literature sources*, *or contact authors due to resource limitations*. *EndNote X9TM was used to manage references*.*”* [55]

Example 3.

**Fig 2:**
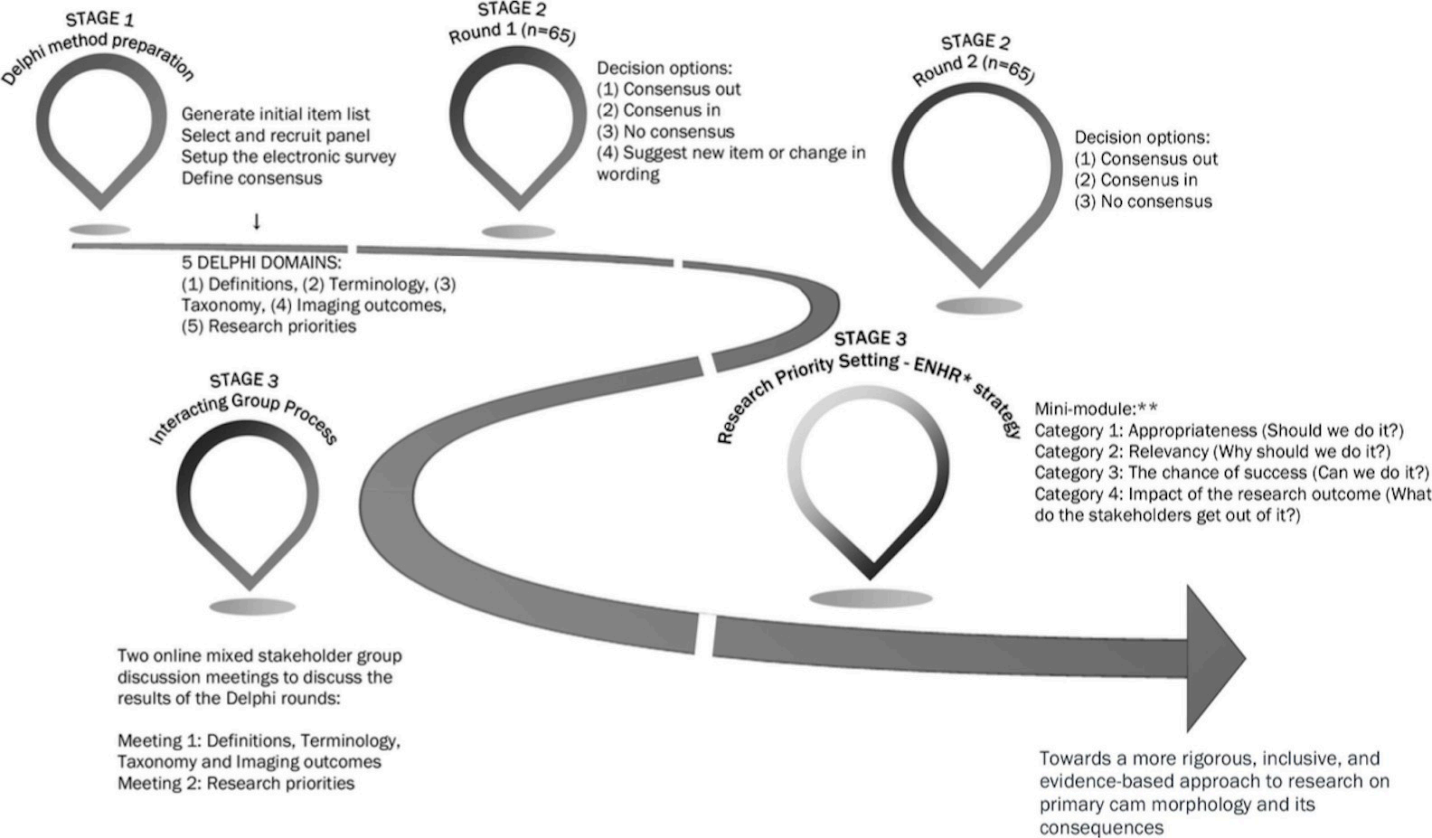
Figure by Dijkstra et al. *Br J Sports Med*. 2022;57:325–341 (DOI: 10.1136/bjsports-2022-106085). Permissions granted by the Copyright Clearance Center at *BMJ* (licensed type CC-BY-NC-ND 4.0) and with the permission of the authors [58].

“*Four search strategies were devised with the aid of validated ‘search blocks’ for core outcomes and search filters for identifying studies on measurement properties*^*18*^ *and patient-reported outcome measures (PROMs)*.^*19*^ *Search strategies are summarized in Figure 2 and detailed in Supplementary Material 1. MEDLINE and Embase databases were searched using the Ovid advanced search function (Ovid Technologies, Wolters Kluwer, USA) on 17 July 2019.*

#### M8. Describe how any existing scientific evidence was summarised and if this evidence was provided to the panellists

Reporting the way in which the scientific information was summarised and whether it was shared with panellists provides insight into the extent to which the panellists were briefed about the consensus exercise. Panellists likely vary in their areas of specialism, topic expertise, lived experiences (see Glossary for “Expertise/expert” and “Experience”), level of education, and preferred learning styles. Reporting the level of background information provided to panellists helps readers understand whether all panellists had the same starting point (namely, a set of uniform information to support their decisions) or whether they were asked to draw only on their existing opinions and/or beliefs about a topic.

Provision of an interpreted data summary (such as a full systematic review with evidence synthesis or a commentary of existing evidence) rather than raw data may influence whether or not a common understanding of the evidence is shared among the panellists. However, providing higher level reviews may also risk introducing bias when panellists are subsequently asked to vote on any proposed recommendations. The decision about whether to provide information should be based on the level of familiarity panellists have with the subject matter (bearing in mind that no gold standard exists), and we recommend that authors report their choice of summary (or no summary) accordingly.

In **Example 1**, the authors explain the evidence synthesis process, the extent to which panellists were given interpreted (GRADE) assessments of the data versus links to the primary source documents, and the platform/format with which information was made available to panellists. The authors of **Example 2** state that relevant evidence was made available to panellists throughout the project, via an online platform and on-demand webinars. However, Example 2 is partially adherent as it misses out detail on how the information in the webinars was summarised and whether it linked directly to the voting options provided to panellists.

To fulfil the requirements of item M8, we recommend that authors:

➢ State **how evidence was summarised** for use in the consensus exercise
➢ Report **whether** the summarised materials were **provided to the panellists**

Example 1.

“*Before the face-to-face meeting*, *the statements were converted to specific patient population*, *intervention*, *comparator and outcome (PICO) questions by the two nonvoting methodologists (F*.*T*., *G*.*I*.*L*.*)*. *The overall certainty of evidence (CoE) [*…*] was graded as very low*, *low*, *moderate or high*. *GRADE evaluations for each statement were provided before the consensus meeting and discussed during the consensus meeting [*…*] The consensus process was facilitated by the CAG [Canadian Association of Gastroenterology] via a web-based consensus platform (ECD Solutions*, *Atlanta*, *GA)*. *The platform allowed consensus participants to review results of the initial literature searches and select and link the references to specific statements*. *Copies of the selected references were available to all members of the consensus group [*…*] At the 1-day consensus meeting*, *evidence for each of the PICO questions was presented*.*”* [57]

Example 2 (partially adherent — 1/2 elements)

“*Panel information pack*

*All panel members had access from the outset of the project and throughout the Delphi process*, *to relevant study material*, *including recorded presentations of the first 8 webinars of the Oxford-Aspetar-La Trobe Young Athlete’s Hip Webinar Series (online supplemental file 4*: *webinar series agenda)*.*”* [58]

#### M9. Describe the methods used and steps taken to gather panellist input and reach consensus (for example, Delphi, RAND-UCLA, nominal group technique)

*If modifications were made to the method in its original form, provide a detailed explanation of how the method was adjusted and why this was necessary for the purpose of your consensus-based study*.

There are numerous examples of formal and informal methods designed to measure or generate consensus. **Table 1** provides just some examples of consensus methods available. A single method of assessing or encouraging agreement may be used, or methods may be combined. For instance, the Delphi method could be used to involve multiple participants from diverse backgrounds before the initiation of an in-person nominal group technique to finalise recommendations. There is no gold-standard method to achieve consensus, but it is important that any methods used are clearly and transparently reported, especially if they have been adapted, to ensure readers understand the process and can judge potential sources of bias.

If consensus developers decide to modify an established consensus method, a clear rationale should be presented. For instance, the three central tenets of the Delphi method are anonymity, iteration, and controlled feedback (See Glossary for “Iteration” and “Anonymity”). Removal of any of these steps can introduce bias (for example through the removal of anonymity) or lead to premature agreement on topics that have not been fully explored (for example not allowing for iteration between rounds does not allow panellists to reconsider their position in the light of new evidence). It is therefore imperative for the reader to be aware of this information.

All three examples offered for M9 meet all of the elements recommended. **Example 1** outlines the method chosen and the reason that it was selected, as well as modifications to the method that supported the outcomes of the consensus exercise. **Example 2** provides an example of modifying the traditional Delphi method. Finally, **Example 3** details an alternative method of consensus (group concept mapping), includes the software used to facilitate use of the method and details the steps involved to reach agreement.

To summarise, we recommend authors:

➢ **Name** the method chosen to measure and/or develop consensus
➢ **Detail all steps taken to get to agreement**. This becomes particularly important if no recognised method is used (namely, the consensus is via informal agreement). In cases where no formal method is selected, a rationale should be provided
➢ **Report modifications** of existing consensus methods alongside **why** the alteration was necessary. For example, the Delphi method starts with an idea generation/proposal round using panellists to provide suggested statements but is often modified to include a first round vote on statements generated by a systematic or scoping review

Example 1.

*“This study used a virtual nominal group technique (vNGT) (Potter et al., 2004), and included researchers and research clinicians from multiple professions who specialized in the study of contextual effects research. We elected to use a vNGT versus a Delphi method because the vNGT allows real time connections between participants, immediate feedback and flexibility when sharing ideas, greater discussion in the later stages of consensus development-thus improving refinement of ideas (Cantrill et al., 2011), all in a shorter time span […]*

*Modifications of a five round NGT are not uncommon and may be warranted when working with complex populations or topics that require maturation before final evaluation (McMillan et al*., *2016)*. *If consensus voting does not identify a clear ranked winner*, *a sixth round*, *which includes re-voting on the top ranked choices*, *can be implemented to assure a true consensus choice (Potter et al*., *2004; McMillan et al*., *2016)*. *Our vNGT used a sixth round of voting to identify a clear consensus definition*.*”* [32]

Example 2.

“*In a ‘classical’ Delphi*, *the first round is unstructured*, *allowing free scope for experts to elaborate on issues they deem important*. *Commonly*, *however*, *this round is structured to save time and effort for both the monitor team and the panellists*.^*6*^

*We assumed that interpretation differences amongst panellists could exist with respect to the items involved*, *and that these could thus affect the discussion of the main research question in a negative manner*. *To avoid such miscommunication on items*, *we added Part 1 prior to focusing on the main research goal in Part 2*, *with both parts consisting of several rounds*. *In Part 1 we combined the collection of all relevant items from the literature and the panel with reaching consensus among panellists regarding the definition of these items*. *Additionally*, *adding a consensus path before introducing the main research question allowed the panel and the monitor team to familiarize themselves with the method and the online survey tool*. *Such clear definitions will also aid interpretability of the results for others*.*”* [59]

Example 3.

“*Group Concept Mapping (GCM)*, … *was used as the method via Concept Systems GroupWisdom™ software*. ^*29*^ *GCM is a participatory mixed‐methods approach*, *where qualitative elements (item generation*, *sorting*, *labelling*, *and rating) are transformed into quantitative data as an integral process by speciality software to gain consensus on a specific topic of interest from a range of participants*. *The consensus emerges from the data via multidimensional scaling and hierarchical cluster analysis*. *Unlike other commonly used consensus methods (e*.*g*., *nominal group or Delphi techniques) that use several rounds of workshops or email conversations*, *only one round of data structuring is involved where participants independently sort statements*, *label each cluster of statements and rate statements to avoid groupthink or peer pressure*.*”* [44]

#### M10. Describe how each question or statement was presented and the response options. State whether panellists were able to or required to explain their responses, and whether they could propose new items

*Where possible, present the questionnaire or list of statements as supplementary material*.

There are many acceptable ways to present topics, statements, or questions in a consensus exercise. The simplest way is just to ask a group to vote in favour or against something by raising their hands in a room. A group might also receive a list of topics electronically, in print, in a meeting room, or even posted by regular mail (as was usual in Delphi studies in the past).

When the consensus exercise is conducted online, the possibilities increase: the web pages might contain just one or several items on which to vote, and the formats of the alternatives for voting, rating or ranking can vary (see the Glossary for more information about the difference between “Rating” and “Ranking”). Participants might be asked to respond in free text (as opposed to closed alternatives), to add comments, or to justify every vote. The study might even start with an open question (as in **Example 1** below). Both offline and online formats for consensus exercises often allow participants/panellists to suggest new items/concepts that can be considered by the full panel either concurrently (for example, in a meeting) or at a future voting round (for example in the traditional Delphi method).

The way a statement or question is presented to a participant can influence their understanding and bias their response. Therefore, readers must be able to see the questions asked of panellists to determine whether they were leading questions or whether they were designed to avoid bias. Authors are encouraged to include details of any methods implemented to prevent the introduction of bias in their questions, or the leadership of any in-person meetings. If an in-person method was selected, then transcripts of the discussions may be included as supplementary material to help readers assess how discourse was managed, and how responses were managed among the panel and any invited facilitator(s) (see Glossary for “Facilitator/facilitation”, “Mediator/mediation”, and “Round robin”).

The examples below follow ACCORD recommendations on what to report regarding this item. Was it easy to respond? Was there space and time for people to express themselves? How much and what kind of information was offered to participants to support them before or during the process? In consensus meetings in person, was there someone to moderate or organise the discussion, as shown in **Example 1** (below). When participants were given a certain number of alternatives to choose, were these clearly labelled (e.g. ‘10 meaning good, 1 meaning bad’)? And was there an intermediate alternative (as recommended by the Likert method), to allow people to be in doubt (which is usually achieved by using an odd number of alternatives)? (See Glossary for “Likert scale”).

**Example 2** demonstrates how a moderator can be used to present questions and control group interaction using the nominal group technique. All these details are useful for the reader.

To understand how panellists could interact with the system (whether in a questionnaire or meetings), the ACCORD item M10 is a reminder to authors to include:

➢ **Questions or statements or proposals formulations** alongside the **format of the response** allowed
➢ Explanation about **whether participants** were allowed or asked to **explain or justify** each of their responses
➢ Information on whether panellists were given **space to suggest new ideas,** and how
➢ The **initial questionnaire**, **list of statements** or **meeting prompts** as supplementary material

Example 1.

*“In round 1*, *the panel was invited to provide free-text responses to the following open question*: *‘What are the important factors to consider when choosing an analgesic for the relief of trauma pain in the pre-hospital*, *emergency room or hospital (i*.*e*. *critical care on the wards and rehabilitation) settings*?*’*. *Responses were grouped into similar themes by the independent administrator; these were then checked*, *revised and consolidated by the co-Chairs to produce a set of agreed factors for use in round 2*. *In round 2*, *the expert panel was asked to rank the importance of each factor using a five-level anchored Likert scale (not important*, *slightly important*, *important*, *very important*, *and extremely important)*. *Importance rankings were compiled by the administrator*, *with the co-Chairs providing expert guidance and advice*. *If more than 75% of the expert panel rated a factor as being important*, *very important*, *or extremely important*, *it was classified as being a ‘provisionally important’ factor to consider when choosing analgesics for the relief of trauma pain; these factors were taken forward into round 3*. *The remaining factors were retained as ‘additional’ factors to consider*. *All results from round 2 were shared and agreed with the co-Chairs for review and validation*, *before initiating round 3*. *In round 3*, *members of the expert panel were asked to rate their level of agreement with the provisionally important factors identified in round 2*. *A five-point pivoted Likert scale was used (0*, *strongly disagree; 1*, *disagree; 2*, *neither agree nor disagree; 3*, *agree; and 4*, *strongly agree)*. *Responses to round 3 were reviewed and validated by the co-Chairs*, *who provided expert guidance and advice*.*”* [60]

Example 2.

*“A skilled moderator (SHM) with expertise in consensus methods led the session in October 2017. To begin, the lead author (CT) summarised the project to ensure that participants clearly understood the study rationale. After the moderator explained NGT (nominal group technique) procedures, participants were asked: ‘What items should be included in a tool to measure the quality of an eConsult?’ Each participant had 15 min to privately write down items. Using a flip chart, a research assistant transcribed one item from each participant in a round-robin format until no items remained. The moderator facilitated group discussion of each item; similar items were combined where appropriate.*

*The lead author then presented a summarised literature review on existing assessment tools for consultation letters (Table 1)*. *This review was conducted to identify studies examining the use of educational instruments to improve specialist-to-PCP written communication*. *[…] We presented the summarised literature review after the round robin to avoid biasing participants during initial item generation*. *A final review of items allowed participants to add*, *combine or remove items as guided by group discussion*. *The research assistant compiled and organised all items onto paper documents that each participant used to anonymously rank each item on a nine-point scale*: *1–3 not essential*, *4–6 neutral*, *7–9 essential to include*.*”* [61]

#### M11. State the objective of each consensus step

*A step could be a consensus meeting, a discussion or interview session, or a Delphi round*.

M9 guides authors to identify the method and any modifications made to the selected method of assessing or achieving agreement. In M11, ACCORD recommends highlighting all the steps taken to reach a consensus. Directly reporting each step will help the reader to see the rigour of the consensus development process.

**Example 1** illustrates a simple approach to describing the steps involved in the Delphi method. **Example 2** is a more detailed instance detailing the steps from a single meeting (using the nominal group technique). Finally, **Example 3** demonstrates an alternative way to present the steps as a flowchart (see **Fig 2**).

We recommend authors:

➢ Clearly report the **steps taken to achieve consensus**, this may be via multiple voting rounds, multiple meetings, or other forms of aggregated group feedback
➢ Explain any **additional steps after the consensus exercise**, especially if recommendations were modified after the final agreement of the panel (for example by the steering committee prior to publication)

Example 1.

*“The PC-COS project comprised three stages*: *(1) a literature review to identify post-COVID-19 outcomes for consideration by the participants; (2) a two-round online modified Delphi consensus process to rate the importance of the selected outcomes for a COS; and (3) an online interactive consensus meeting to review and agree upon the final COS*.*”* [40]

Example 2.

*“Stage one (Introduction and Explanation): An introduction and welcome to all participants with an explanation of the purpose and procedure of the workshop.*

*Stage two (Silent Idea Generation): The question was introduced to the participants: “What is a working definition of contextual factors”?*

*Stage three (Sharing Ideas): During Stage three, each participant introduced their definitions that were recorded on Google documents.*

*Stage four (Group Discussion): Participants were invited to seek verbal explanation or further details about any ideas that were produced during stage three.*

*Stage five (Voting)*: *During stage five*, *and after the week of modifying or deleting their own contributions*, *vNGT participants were allowed to ‘rank order’ the definitions generated during stage four*.*”* [32]

Example 3.

#### M12. State the definition of consensus (for example, number, percentage, or categorical rating, such as ‘agree’ or ‘strongly agree’) and explain the rationale for that definition

The definition of consensus or ‘when agreement exists’ among the selected panel is extremely important. In most instances where agreement is sought it would be normal to expect a variety of opinions. For those reading the consensus exercise to understand fully what level of agreement has been sought for each statement, an *a priori* level of agreement should be reported (see Glossary for “Consensus threshold”). This is most often expressed as a percentage of the number of panellists in agreement, but can take multiple forms, for example a certain proportion of people rating ‘strongly agree’ on a categorical scale.

Currently there is no gold standard for where to set the definition of consensus; indeed, it may vary from project to project. For instance, a consensus exercise to agree the infection control policies in a surgical environment – or an exercise in any context where there is a high risk of severe negative outcomes – may seek a high level of agreement. Whereas, consensus sought on which research priorities should be addressed may allow for a lower level of agreement due to the lack of immediate risks (that is not to say there are no future risks in this scenario, only that they are less immediate), and the improbable event that all parties (who may have their own vested interests) will come to an unanimous agreement.

The level of agreement may also be influenced by the number of panellists: it is much easier to get unanimity among a panel of five than among a panel of 95 people. This inherent variation between research questions is why it is important to explain the rationale for selecting a specific threshold designated as ‘agreement’.

**Fig 3:**
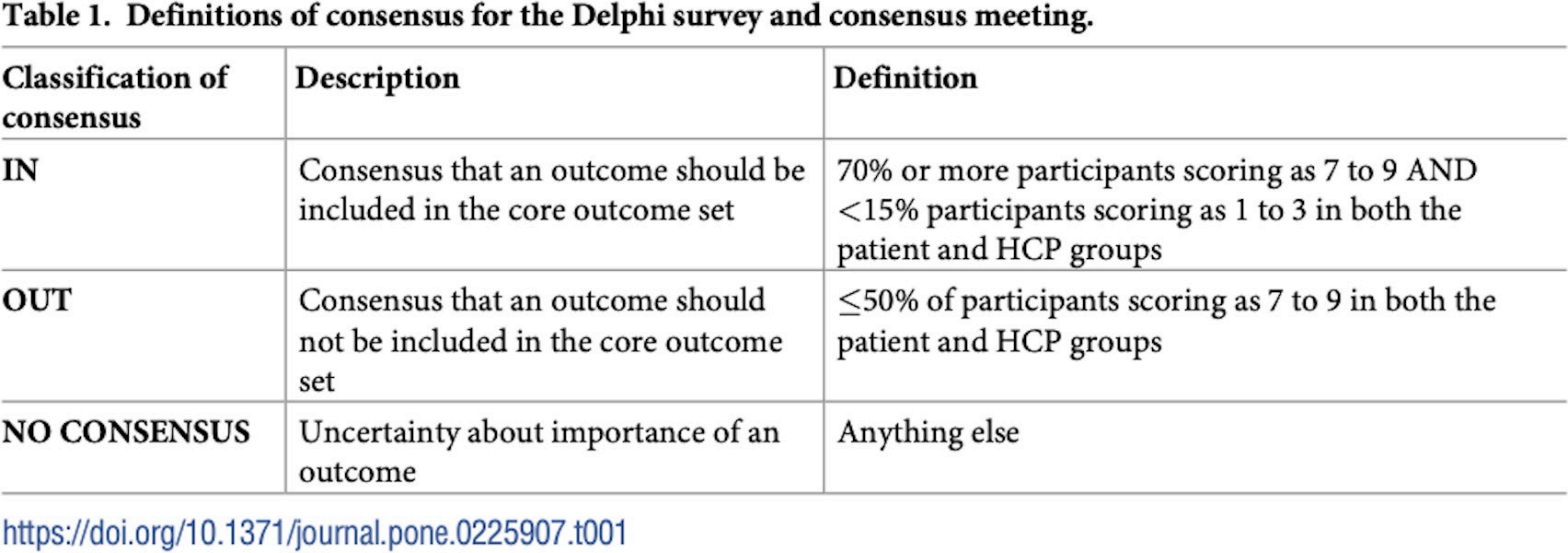
Table by Srikandarajah et al. *PLOS One*. 2020;15: e0225907 (DOI: 10.1371/journal.pone.0225907). This work is licensed under a CC-BY 4.0 licence [53].

**Example 1** uses a percentage agreement to represent consensus, this time measured following the Delphi method. The example helpfully includes information on dissent (see Glossary for “Disagreement/dissensus”), by summarising that there had to be <15% of participants disagree with a classification or recommendation. This example also shows the use of a table to summarise ‘consensus IN’, ‘consensus OUT’ (otherwise known as consensus disagreement) and ‘No consensus’ (see text and **Fig 3**). **Example 2** illustrates a descriptive way of explaining the definition of consensus. Example 2 is the standard procedure for measuring agreement using the RAND/UCLA appropriateness method.

We recommend that authors report:

➢ The **rating/ranking scale** used, for example whether people were asked to assign a number, percentage, or categorical rating such as ‘agree’ or ‘strongly agree’
➢ Whether **dissenting views** affected the ability to declare consensus and at what level dissent was consider significant (for example when one person disagreed, or when X percentage disagreed with the rest of the group)
➢ The **level** at which the panellists were **considered to be in agreement**, namely when agreement was sufficient to call a ‘consensus’

Example 1.

*“We have adopted the “70/15” consensus definition in the protocol*, *which was used successfully in other COS [core outcome set] studies*^*20*,*21*^ *for inclusion of an outcome in the COS*. *However*, *it was partially revised for “consensus out” due to the study team’s experience from other studies where outcomes were rarely voted 1–3 not important and reach criteria for exclusion after the Delphi survey*… *the final definitions of consensus that were used are in Table 1*. ”[53]

Example 2.

*“According to the RAND/UCLA Appropriateness Method*, *the panelists ranked the appropriateness of each treatment on a nine-point scale*, *in which a score in the range 1-3 is considered ‘inappropriate’*, *4–6 ‘uncertain’*, *and 7–9 ‘appropriate’*. *We then pooled these scores to generate a median appropriateness score for each treatment according to patient sub-phenotype*. *In addition*, *according to RAND/UCLA methodology*, *we classified the presence of ‘disagreement’ among the votes for a treatment modality if greater than one-third fell in the opposite tertial to the median score [e*.*g*., *a vote was considered in “Disagreement” if it received an “Appropriate” median vote (≥7) with five of 13 members voting “Not appropriate”(≤3)]*. *Finally*, *we classified a treatment as “Appropriate” if it received a median score of 7 without disagreement*. *A treatment was classified as “Not appropriate" if it received a median vote of 3 or lower without disagreement*. *A treatment receiving a score between 3 and 6*, *or a treatment with disagreement*, *was classified as “Uncertain”*.*”* [36]

#### M13. State whether items that met the pre-specified definition of consensus were included in any subsequent voting rounds

In consensus exercises that involve multiple rounds of voting, statements that were ‘agreed’ in the first round of voting (that is, those that met the agreed consensus criteria) may be treated as final and be excluded from subsequent voting rounds, or they can be retained for further voting. Authors should report whether those leading the consensus exercise decided to remove an item that was excluded by panel vote, or to submit it to another voting round.

The retention of items that have met consensus criteria in subsequent rounds allows researchers to assess the stability of agreement (explicitly, whether opinions are settled or moving in response to additional ideas or feedback) [62] (see Glossary for “Stability”). The aggregate opinion of the group can remain stable even if there are fluctuations in the opinions of some individuals within the group. Stability of the consensus opinion over time, or over multiple voting rounds, can indicate a more robust consensus decision.

In the following examples, **Example 1** specifies that only items that had not reached the defined threshold for consensus were included in subsequent voting rounds (See Glossary for “Consensus threshold”). In contrast, the authors of **Example 2** explain that all statements were retained across all voting rounds and rescored in each round. **Example 3** includes a detailed explanation of the definition of consensus used for the exercise, as well as how it and the stopping criterion were applied in their approach.

Therefore, when consensus exercises require multiple rounds, it is recommended that authors include:

➢ Whether **items that met the threshold for consensus** in one round were removed or included in subsequent rounds
➢ Whether any specific ‘**stopping criterion**’ was applied to either exclude items from subsequent rounds or to end the consensus exercise

Example 1.

*“If the proportion of participants either agreeing or disagreeing with a statement did not exceed 66%*, *that statement would be revised according to the feedback received and another survey initiated that included only the statement(s) not reaching consensus*. *This process would be repeated*, *with the statements being revised*, *until consensus was reached for every statement*. *For those statements that received ≥25% disagreement during Step 2 (i*.*e*. *≥25% participants voted ‘disagree’ or ‘absolutely disagree’)*, *the reason(s) for this disagreement were explored further*.*”* [63]

Example 2.

*“The Delphi study steering committee retained all statements between rounds 1 and 2 to enable participants to re-score every statement after considering feedback from round 1*. *Acknowledging that certain statements might be more relevant to some panel members than others*, *stakeholders were given the choice not to score a specific statement*.*”* [58]

Example 3.

*“Defining consensus and stability*

*We used the RAND criteria for agreement to define consensus^46^ […] Consensus was defined as 80% of ratings within the 3-point tertile of the overall median. The lower tertile (1–3) represents scores that are ‘not at all’, the middle tertile (4–6) represents scores that are ‘somewhat’, and the upper tertile (7–9) represents scores that are ‘very’ important, actionable, and/or necessary. To be included in the final set, quality statements and PMs needed to reach consensus in the upper tertile (i.e., overall panel median of 7 to 9, with 80% of ratings within the 3-point tertile of the overall median). Those that achieved consensus just below a priori thresholds were considered during qualitative data analysis and interpretation to determine justification for potential inclusion.^37^*

*A measurement of stability was used as a stopping criterion for the Delphi process*. *This was defined as the consistency of responses between successive rounds (i*.*e*., *no meaningful change)*.^*44*^^,^^*45*^^,^^*48*^ *Meaningful change was defined as a median change between tertiles and a greater than 15% change in the percentage of participants whose scores changed tertiles*.^*45*^^,^^*48*^ *[…]*

*For the overall study*, *the criterion to stop the Delphi process was defined as no meaningful change in scores between the current and preceding round on at least 75% of quality statements and PMs assessed*. *Additionally*, *criteria for PM removal were considered after the second round*. *To be removed from the process*, *a PM’s scores must have shown no meaningful change from the previous round*, *and there must have been consensus that the PM is not necessary (i*.*e*., *overall panel median of 1 to 3*, *with 80% of ratings in the lower tertile)*.*”* [64]

#### M14. For each step, describe how responses were collected, and whether responses were collected in a group setting or individually

Some consensus exercises may wish to preserve participant anonymity and minimise the potential for group influence (for example, peer pressure — see Glossary entry for “Dominance/peer pressure”). They therefore avoid open group discussions when collecting response data. Other exercises may use data collection platforms that make participants’ (pooled) responses visible to the wider group while maintaining anonymity using pseudonyms. For consensus exercises that seek input from a geographically diverse group of participants, a method of remote data collection is required, and, with the increase of video-conferencing software, consensus meetings can be held across multiple time zones without the need to travel. There are no one-size fits all approach to data collection; the decision on how to collect responses will be determined by the method, scope, and resources of the consensus exercise. Whatever the selected methods, reporting them allows readers to consider the potential for bias, power dynamics and/or group influence to have shaped the consensus findings.

The authors of **Example 1** report the format of each data collection stage, the software/platforms used and whether individual or group/collaborative responses were required at each step of the exercise. In **Example 2**, individual responses were sought from panellists. In **Example 3**, the authors describe the rounds of a Delphi exercise, reporting the format of each, and the supporting software used. It is implicit in the description of the approach that the first two rounds were individual, and that the final round was conducted in a group setting. This example, however, fails to report sufficient detail about the way in which responses were collected in the third and final round.

To fulfil all requirements of item M14, we recommend that authors:

➢ Provide details on **how responses were collected**, such as via an online survey, online voting platform, over the phone or face to face
➢ Specify whether responses were collected **individually** or in a **group** setting

Example 1.

*“Stage two (Silent Idea Generation): The question was introduced to the participants: “What is a working definition of contextual factors”? All participants were asked to create a list of ideas that come to mind when considering the question and to place these ideas on a shared Google document. During this stage, all participants were asked not to consult or discuss ideas with each other. A total of 10 min was provided for each participant to create his or her selected definitions. […] Unique to this vNGT [virtual nominal group technique], participants had up to 1 week to modify or delete their own contributions or request edits to another definition that they did not generate. We elected to provide additional time to edit each person’s definition, since the concept is complex and since there were a variety of definitions presented in Stage two and three, which were further discussed and modified in Stage four.*

*Stage five (Voting)*: *During stage five*, *and after the week of modifying or deleting their own contributions*, *vNGT participants were allowed to “rank order” the definitions generated during stage four*. *Rank ordering was performed using a Qualtrics survey and a “ranking” function*. *In this survey*, *each NGT participant ranked all 12 definitions from 1 (top choice) to 12 (lowest choice)*.*”* [32]

Example 2.

***“****An online survey tool was used (SurveyMonkey.com) with a clear lay-out that was identical in all rounds (Fig 1). We presented one item per page, including a statistical summary of the former round, and anonymized remarks from prior rounds. Each member of the panel received an individual invitation for each survey. A survey could be closed and continued whenever the panellists wanted this.”* [59]

Example 3.

*“The first two rounds of the Delphi were online surveys administered using Surveylet*. *Surveylet is a purpose-built platform for developing and administering Delphi surveys*.^*19*^ *To start with*, *the Delphi participants were presented with an initial set of 17 potential open science practices to consider that were generated by the project team based on a discussion*. *Round 3 took the form of two half-day meetings hosted on Zoom*.^*20*^*”* [65]

#### M15. Describe how responses were processed and/or synthesized

*Include qualitative analyses of free-text responses (for example, thematic, content or cluster analysis) and/or quantitative analytical methods, if used*.

The choice of quantitative or qualitative methods for processing responses will be dictated by the consensus method being used, the format of statements or questions, and the overall aims of the consensus process. For instance, seeking a detailed and harmonized agreement of patient experiences of healthcare is likely to be more meaningful when grouped using a thematic analysis than by simply providing an agreement score or percentage. There is no gold standard method, but at the protocol or planning stage a decision should be made about how to deal with group feedback, and how responses will be utilised to inform the final report.

**Example 1** demonstrates how a qualitative method can be used to analyse data from a Delphi-survey (via ‘thematic analysis’). **Example 2**, describes a mixed-methods approach to consensus data synthesis. Finally, **Example 3** is an alternative mixed-methods approach to assessing the data generated through the modified Delphi method. These three examples demonstrate there are many acceptable ways of synthesizing consensus-related data, and that the decision should be made according to the individual context of the consensus exercise. ACCORD simply recommends that the choice is reported clearly.

Example 1.

*“In R1 and R2*, *to enable in-depth exploration of the quantitative findings*, *panellists were provided with the opportunity to make further comments as they saw fit*, *through open questions*. *Thematic codes were identified using Framework Analysis*,^*46*^ *a matrix-based method for ordering and synthesising data*. *The analysis was conducted by DS*. *Quality checking of the coding process and reduction of coding bias were ensured by AJ*, *who reviewed 10% of the qualitative data*. *First*, *data were reviewed inductively to identify recurring themes and concepts raised by panellists*. *These were coded and formed the initial major and subthemes; additional codes were then incorporated through an iterative process involving DS and AJ*. *The thematic framework was further refined before being applied systematically to the whole dataset*. *This process facilitated the identification of any inconsistencies in coding*, *which were subsequently discussed and reconciled*.*"* [33]

Example 2.

*“We entered and stored data using the DelphiManager electronic software tool and created Excel spreadsheets.^26^ We calculated descriptive statistics for each statement and stakeholder group for example, summary scores, ranges, percentage scoring for each statement ‘not important/disagree’ (score 1–3), ‘important but not critical/neutral’ (score 4–6) and ‘critical/agree’ (score 7–9). Specifically, we reported, per stakeholder group, the median and IQR for each statement between each round (online supplemental file 5)[…]Qualitative analysis*

*The lead investigator (HPD) immersed himself in the details of participants’ comments provided during Delphi rounds*, *Interacting Group Process and ENHR [Essential National Health Research] ranking exercise*.^*44*^ *After developing a framework based on recurrent and important themes*, *the free text comments were grouped into categories*, *iteratively discussed between the lead investigator and second author (SMA)*. *The lead authors (HPD and SMA) then undertook thematic analysis to identify*, *group and agree on common threads within these categories*, *further refining themes and subthemes*.^*45 46*^ *We provided summarised feedback of quantitative and qualitative open responses to panel members during Webinars 10 and 11 of the Oxford-Aspetar-La Trobe Young Athlete’s Hip Webinar Series*. *The two webinars preceded the online synchronous mixed stakeholder group discussions*.*"* [58]

Example 3.

*“Across the three rounds*, *we ran frequencies of all statements and recommendations (Supplementary Discussion 2); the proportion who selected ‘not qualified to respond’ is reported in the data tables but removed from the denominator to calculate levels of agreement/disagreement from the relevant sample*. *The team then analysed the extensive qualitative data (that is*, *open-ended text-box comments)*. *Specifically*, *comments were first reviewed individually by at least three core group members (J*.*V*.*L*., *co-chair; D*.*R*., *methodologist; and C*.*J*.*K*.*) and an additional co-author (T*.*M*.*W*.*)*. *For each data collection round*, *comments were then discussed in online review meetings*, *including at least three core group members and an additional co-author*. *After review and discussion*, *comment suggestions were incorporated into statement and recommendation revisions for subsequent rounds*. *A supermajority of core group members (28 out of 40; 70%) participated in the online consensus meeting*, *which permitted in-depth breakout-group discussions on salient issues from R1 and R2 informing R3 revisions (Supplementary Discussion 3)*. *Quantitative analysis of the final R3 results involved assigning each statement and recommendation a grade to indicate the level of combined agreement (agree + somewhat agree)*, *using a system that has been used in other Delphi studies^139–141^ in which ‘U’ denotes unanimous (100%) agreement; ‘A’ denotes 90%–99% agreement; ‘B’ denotes 78%–89% agreement; and ‘C’ denotes 67%–77% agreement*. *Although all statements and recommendations exceeded the standard supermajority minimum of ≥67% combined agreement for consensus*, *we highlighted those with <67% for ‘agree’ alone for further analysis*. *Statements and recommendations were analysed using Fisher’s exact tests in Stata (v*.*16) to assess differences in agreement by the following panellist characteristics*: *income level (high income versus low- and middle-income) for country of birth and country where currently working*, *primary sector of employment and primary field of employment (Supplementary Discussion 2)*. *The use of the terms combined agreement and combined disagreement are presented in the results*.*"* [41]

#### M16. Describe any piloting of the study materials and/or survey instruments

*Include how many individuals piloted the study materials, the rationale for the selection of those individuals, any changes made as a result, and whether their responses were used in the calculation of the final consensus. If no pilot was conducted, this should be stated*.

Piloting is an important step in a study design. Pilot studies can be used to test the applicability and feasibility of: questionnaires; meeting formats; recruitment strategies; the quality of materials; and other parts of the study design. Testing these materials and procedures on a small scale before starting the consensus process can give the researcher valuable insight about what works and how.

As many consensus processes apply structured questionnaires, researchers can check whether the questions are correctly formulated and understandable by participants. This can best be done by running a number of surveys using the materials before the real exercise starts, allowing time for corrections and adjustments. Modifications after piloting can go from simply replacing problematic words to rearranging the order of questions or response alternatives, labelling them, increasing or decreasing the time to respond, modifying recruitment messages, and others [66,67].

Completing a pilot shows that the researchers were keen to apply the most robust methods possible in their consensus exercise [68]. Another issue to consider, and to report, when piloting is whether the individuals participating in the pilot are also engaged with the ‘real’ research conducted afterwards, in this case, the consensus. This is relevant for data analysis and interpretation, as some individuals would have been more exposed to the materials, with possible bias.

The ACCORD guideline recommends reporting:

➢ **which** materials were piloted,
➢ by **how many** participants,
➢ the **rationale** for the inclusion of the people involved,
➢ **what** was done as a result, and
➢ whether the piloting results were **used in the calculation** of the final consensus.

**Example 1** below mentions that a questionnaire was piloted, who the participants piloting it were, and how many of them there were. It also explains that the questionnaire was edited as a result of the pilot, but it does not explain what these modifications were nor if the 11 people piloting the material were included in the final consensus. In **Example 2**, we know that three experts from different countries were selected to pilot the paper version of the material. Example 2 also provides a succinct summary of the changes made as a result of the pilot test. However, it fails to specify why the experts were chosen or whether these three people (or their results) were included in the final consensus panel.

We were unable to locate an example in the existing literature that touched on all five of the elements recommended in M16. Authors are encouraged to pay particular attention to each of the five elements should they decide to pilot materials in their consensus process. No examples found described the rationale for selection of their respective pilot participants.

Example 1 (partially adherent — 3/5 elements).

*“A pilot study to test the R1 survey questionnaire*. *As a strategy to reduce attrition*,^*24*, *25*^ *careful attention was paid to the content and layout of the invitation email*, *the survey layout and the clarity of questions*. *Piloting was conducted with academic (n=6) and user-researchers (n=3) and members of our Patient Advisory Group (n=2)*. *Language*, *question type and questionnaire formatting were edited in response to participant feedback*.*”* [33]

Example 2 (partially adherent — 3/5 elements).

*“The paper version was pilot tested with three palliative care experts from three different European countries (Germany*, *the U*.*K*., *and Slovenia) to affirm the comprehensibility of the questionnaire and the usefulness of the response options*. *Based on the pilot test*, *the introductory text was structured more clearly and the language was simplified*. *The wording of some statements was modified*, *and a five-point scale was chosen as the most preferred response option*. *In response to initial feedback about the length of the questionnaire*, *a status bar indicating completion progress and the option to complete the questionnaire in several steps were added to the instrument*.*”* [69]

#### M17. If applicable, describe how feedback was provided to panellists at the end of each consensus step or meeting

*State whether feedback was quantitative (for example, approval rates per topic/item) and/or qualitative (for example, comments, or lists of approved items), and whether it was anonymised*.

Many consensus methods require aggregated group feedback so that fellow panellists can assess their position in relation to their peers (as demonstrated in **Example 1**). This is often presented as quantitative data (group mean/median ratings and the range of responses) and/or qualitative data (comments from panellists or aggregated themes). Feedback may also take the form of simple verbal feedback or presentations in a consensus conferences, or summary notes in the case of smaller group meetings.

Providing a record of the feedback given to support consensus measurement or development allows the reader to assess the iterative nature of the process. This has the potential to demonstrate where key points of convergence or contention arose, and how feedback may have influenced this process.

**Example 2** demonstrates how qualitative feedback was incorporated between rounds, but the authors could have been clearer about whether all responses were visible to participants. **Example 3** describes how both verbal and visual (on-screen) feedback was provided to panellists as part of the round-robin stage of consensus development (see Glossary for “Round robin”).

We recommend that authors:

➢ Report the **form** of feedback (verbal or written)
➢ Identify whether feedback was **quantitative, qualitative or a mix**
➢ **Summarise or provide copies of the** feedback where appropriate, taking care to anonymise information in your report where necessary.

Example 1.

*“Each item in the questionnaire was displayed with the panellist’s prior response in Questionnaire 2 and the mean ± SD of the group’s response in Questionnaire 2*. *This strategy has been used in previous Delphi studies to help reinforce consensus*.^*26–30*^*”* [70]

Example 2.

*“All rounds allowed for overall comments at the end of the survey*, *and the researchers reviewed 1*,*409*, *755*, *and 188 comments associated with the statements in R1*, *R2 and R3*, *respectively*, *and 1*,*025 and 2*,*156 comments associated with the recommendations in R2 and R3*, *respectively*. *Summaries of changes based on panellist input from a previous round were available in text boxes next to each statement and recommendation in the subsequent round*.*”* [41]

Example 3.

*“*…*each participant introduced their definitions that were recorded on the Google documents*. *This document was shared on the screen so that all participants can see the list in real time*. *This stage continued in a round robin format until all ideas had been presented*. *No debate or discussion occurred at this stage*.*”* [32]

#### M18. State whether anonymity was planned in the study design. Explain where and to whom it was applied and what methods were used to guarantee anonymity

Anonymity can encourage panel members to express their views more freely, less affected by the urge to conform to or refute others’ views (see Glossary for “Anonymity” and “Dominance/peer pressure”). Anonymity also gives space for the expression of some opinions that could be rejected based on who proposed the view. The other side of anonymity is that people are not held accountable for individual votes or decisions, so anonymity may not always be desirable. It is crucial to report whether anonymity was applied, when, and how.

Anonymity is one of the principles of Delphi surveys but does not only apply to Delphi exercises. People gathered in a physical room and introduced to each other might still use electronic devices (as shown in **Example 1** here) to vote, with their individual decisions not attached to their identities, or even have their identities coded and not revealed to each other (as shown in **Example 1**). An administrator can also know the identities but hide them from other researchers and panellists, as shown in **Example 2**. In online meetings it becomes even easier, as shown in **Example 3**, where the platform used hides the voters’ identities from anyone participating, including the organisers of the meeting. In these cases, the group knows who is present and participating, but the anonymity is preserved for the final voting, even if individuals expressed verbally in favour or against a given topic.

Anonymity should be reported even by authors not using it in their studies. Using ACCORD item M18, authors will take the chance to explain:

➢ **Whether** anonymity was used,
➢ To **whom anonymity was offered** each consensus step — with a clear statement when it was not,
➢ What **measures** were taken by authors **to guarantee anonymity** in each phase.

Example 1.

*“Results [from the online voting] were compiled by the CAG to ensure voter anonymity*. *At the three-day consensus meeting* … *all voting participants used electronic keypads to record two separate anonymous votes on each statement*.*”* [71]

Example 2.

*“For tracking purposes*, *the administrator knew the identities of responding panellists*, *but no identifying information was shared with the co-chairs or other panel members*. *Panellists remained anonymous to each other throughout the Delphi stages*.*”* [51]

Example 3.

*“During the second meeting of the international expert panel*, *using the anonymous polling feature on Zoom*, *each panelist voted to approve*, *approve with modifications*, *or reject each proposed intervention*.*”* [72]

#### M19. State if the steering committee was involved in the decisions made by the consensus panel

*For example, whether the steering committee or those managing consensus also had voting rights*.

As stewards of the consensus exercise, the steering committee (or those managing/leading the consensus exercise) typically hold knowledge relevant to the topic under consideration (see Glossary for “Steering committee”). However, they may also have the potential to exert (directly or indirectly) undue influence on the panellists’ decision-making or the eventual outcome of the results. Reporting any involvement that the steering committee had in developing the eventual recommendations, other than facilitation (see Glossary for “Facilitator/facilitation”), is therefore valuable to report in the interests of transparency. Steering committee involvement in the decisions made by the panel may include: participation in voting or ranking activities; acting as adjudicators where consensus is in doubt; or reserving final decision-making powers once voting has ceased.

**Example 1** uses a tabular format to disclose the roles of all individuals involved in the consensus exercise; the fact that steering committee members were involved in the voting/decision-making process is clearly reported (requires reader to link to original text). **Example 2** uses a simple text format to disclose that the chairperson had voting rights, and that there were also a non-voting moderator and an observer. Finally, **Example 3** is a statement of the involvement of the co-chairs as well as the panellists in the decision-making process.

To fulfil the requirements of item M19, we recommend that authors:

➢ Report whether **some, or all, of the steering committee** played a role in the panellists’ decision-making process and/or played a part in forming or changing the consensus recommendations
➢ Where steering committee members did play a role, specify the **nature of that role**, such as participating in voting/ranking or adjudication

Example 1.

Please follow DOI link (DOI: https://doi.org/10.1212/NXI.0000000000200124) to view the authors’ **Table 1**, a tabular summary of the participants of a Delphi exercise, and their respective roles. [73]

Example 2.

*“A face-to-face consensus meeting was held in Toronto*, *Canada*, *in February 2018*. *The international consensus group comprised five voting gastroenterologists (including the chair*: *D*.*C*.*S*.*)*, *from Canada*, *the United States and the United Kingdom*. *Other participants included a nonvoting moderator (J*.*K*.*M*.*)*, *the two GRADE experts (F*.*T*., *G*.*I*.*L*.*) and a nonvoting observer*.*”* [57]

Example 3.

*“All members of the expert voting panel and the 2 voting co-chairs voted anonymously on the statements to reach consensus*.*”* [74]

#### M20. Describe any incentives used to encourage responses or participation in the consensus process

*For example, were invitations to participate reiterated, or were participants reimbursed for their time*.

Incentives, such as payment, personal encouragements or promise of reward are sometimes used in consensus research to encourage participation and retention, and to acknowledge panellists’ contributions. As with all types of encouragement, there is potential for financial or in-kind incentives to create a conflict of interest (see Glossary for “Conflict of interest”). It is relevant to note that there is potential for selection bias irrespective of whether individuals are motivated to participate in a consensus exercise for financial, in-kind benefits, or personal reasons. It is, therefore, valuable to be transparent and to give readers a clear understanding of possible biases introduced by the use, of not, of incentives.

In **Examples 1**, the authors describe a process of invitations and timely reminders to encourage participation. **Example 1** also explicitly states, as does **Example 2**, that no financial incentives were offered for participation. Similar to Example 1, **Example 3** reports the use of invitations and considerate reminders to encourage participation.

We recommend that authors report all strategies and incentives used to:

➢ **Encourage** participation in the consensus exercise, financial or not
➢ Improve participant **response rates and retention** throughout the consensus exercise (See Glossary for “Response rate”, “Completion rate”, and “Fatigue/survey fatigue”)

Example 1.

*“After inclusion, panellists received an e-mail with study details and additional message in the week leading up to each round. The link to the survey was sent by e-mail and a text message was sent by phone. When the research team felt it was necessary, panellists received a reminder. This was done on the basis of prior response times of the individual panellists, expected duration of completion and national holidays. Panellists were prompted by one researcher (MG)*.

*Incentives to participate could include being invited to join a selective group and the opportunity to learn from the consensus process*.^*5*^ *We did not pay our experts*, *nor did we give them presents of any kind*.*”* [59]

Example 2.

*“No honoraria were provided to the steering committee or the expert voting panel for participating in this initiative*.*”* [74]

Example 3.

*“Email invitations to individuals were followed up with a reminder after a week*, *to ensure that people had ample opportunity to respond without unduly ‘pestering’ them*. *A non-response to the follow up email was considered a decline to participate*.^*27*^*”* [75]

#### M21. Describe any adaptations to make the surveys/meetings more accessible

*For example, the languages in which the surveys/meetings were conducted and whether translations or plain language summaries were available*.

Consensus exercises often aim to involve a diverse participant group that is broadly representative of the wide range of people who could be affected by the consensus recommendations. As a result, participants may include experts, ’non-experts’ and individuals with varying sociodemographic and professional backgrounds (see Glossary for “Expertise/expert” and “Experience”). In such circumstances, adaptation of the consensus materials and activities may be warranted to ensure they are accessible to all participants. (See Glossary for “Accessibility”.) Adaptions are not required for all consensus exercises but, where relevant, they may take the form of foreign language translations, language simplifications, modifications for individuals with visual or hearing impairments, and the provision of materials and delivery of activities in both physical (hard copy and/or face-to-face) and online (e-surveys, virtual meetings) formats.

All three of the provided examples report some level of language translation to improve the accessibility of the consensus materials used for participants. **Example 1** describes translation of materials by co-researchers for non-English speakers, **Example 2** lists the languages into which materials were translated for each round, and **Example 3** not only reports the languages into which the materials were translated and the translation process used, but also acknowledges limitations of that process. In addition to considerations of language accessibility, **Example 1** also describes adaptations that were made to the data collection method to overcome accessibility issues for some participants associated with online data collection.

Example 1.

*“The original plan was to collect data online only due to Covid restrictions. However, it was found that some participants experienced issues with completing the requested activities online, particularly older people and those who spoke little or no English. Therefore, an offline approach was adapted for data collection. There was no difference in the type of data collected by the two differing modes of participation.*

*For online participation*, *participants either completed the activities independently or with support from the first author over the telephone*. *For offline participation*, *relevant paperwork was posted to participants or co‐researchers*, *who were recruited and trained to recruit and collect data from people in ethnic minority communities*. *Participants completed the requested paperwork on their own or with support from a co‐researcher*. *When collecting data from participants who did not speak English*, *a co‐researcher translated the study information orally*, *and any data collected were translated into English by co‐researchers*. *The same type of data was collected from participants who completed the paperwork alone and those who completed it with support from a co‐researcher*.*”* [44]

Example 2.

*“Round 1 was available in English*, *Chinese*, *French*, *and Spanish and ran for approximately 3 months*, *to enable recruitment of as many participants as possible* … *This shortened list was reviewed by six steering group members (BKS*, *HT*, *PTK*, *NC*, *SKW*, *and TYW)*, *and further consolidated to a list of challenges for round 2*, *which was available in English*, *French and Spanish (all participants answering in Chinese in round 1 were able to complete subsequent rounds in English)*.*”* [76]

Example 3.

*“All five versions were translated into Spanish and Chinese using native-speaking volunteer committee members (C*.*H*. *and Z*.*C*.*)*. *With the assistance of the Spanish Respiratory Scientific Society nursing assembly*, *the Spanish versions were pilot tested with 10 nurses*, *5 patients*, *and 2 caregivers*, *and needed revisions made*. *This process was limited in that no back translations were performed for either Spanish or Chinese*, *and no piloting of the Chinese language version was done*.*”* [77]

### Manuscript section: Results

#### R1. State when the consensus exercise was conducted

**List the date of initiation and the time taken to complete each consensus step, analysis, and any extensions or delays in the analysis**.

Reporting when consensus steps (as outlined in M11) and when the consensus analysis took place can provide valuable clinical and societal context about the temporal influences that may have affected each activity. Understanding where delays or challenges may have occurred may also be instructive for those replicating the work or considering similar exercises.

In the provided examples: **Example 1** reports the start and end date of each Delphi round and also the date that the draft guideline was circulated to participants. **Example 2** describes when each consensus exercise was undertaken and how long each step took, however it fails to explain when the data that arose from the meetings was analysed/synthesised. We have therefore marked Example 2 as partially adherent.

When procedures were planned to happen and when they actually occurred may be different things. In the Results section of the paper, item R1 of ACCORD reminds authors to report:

➢ Start and end date of **each step** (meeting, voting round)
➢ Start and end date when researchers **analysed/synthesised the resulting data**

Example 1.

*“The Delphi process consisted of 2 rounds of survey, response and feedback. The first-round survey (Round 1, March 27, 2019–May 17, 2019) invited participants to score items from the preliminary list and to submit additional reporting items. The second-round survey (Round 2, May 31, 2019–July 12, 2019) provided feedback from the previous round and invited participants to rescore items. […]*

*Post consensus meeting development*

*Following the consensus meeting*, *MC drafted the reporting guideline with guidance from the steering committee*. *The following minor amendments were made*: … *The resulting draft guideline was circulated to all consensus meeting attendees in March 2020*. *All comments and revisions were taken into consideration*, *and the checklist revised accordingly*.*”* [78]

Example 2 (partially adherent — 1/2 elements).

***“****We discussed proposed content and wording of the PRISMA 2020 statement*, *as informed by the review and survey results*, *at a 21-member*, *two-day*, *in-person meeting in September 2018 in Edinburgh*, *Scotland*. *Throughout 2019 and 2020*, *we circulated an initial draft and five revisions of the checklist and explanation and elaboration paper to co-authors for feedback*. *In April 2020*, *we invited 22 systematic reviewers who had expressed interest in providing feedback on the PRISMA 2020 checklist to share their views (via an online survey) on the layout and terminology used in a preliminary version of the checklist*.*”* [79]

#### R2. Explain any deviations from the study protocol, and why these were necessary

*For example, addition of panel members during the exercise, number of consensus steps, stopping criteria; report the step(s) in which this occurred*.

Registering a protocol (a plan) for the consensus approach is encouraged from a research transparency and replicability perspective (as noted in the explanation for item M1). A protocol, however, may be modified after registration for many different reasons, including adaptions necessary to overcome implementation challenges, or revising part of the methods or analytical approach to consider new information that arose after the initiation of the consensus exercise. Reporting deviations from the original protocol/plan allows readers to understand where the approach was adjusted, or if aspects of the approach were not feasible. Transparency about modifications to the intended approach provides insight into the robustness of the final consensus approach and may help inform the design of activities seeking to replicate the work.

In **Example 1**, the authors report which components of their approach were not planned *a priori* and why organic modifications were required. **Example 2** discloses a deviation from the *per protocol* definition of non-consensus (disagreement) for their Delphi survey (see Glossary for “Disagreement/dissensus”). Finally, the rationale provided in **Example 3** for calling off a third round of voting implies a deviation from the intended number of *per protocol* survey rounds.

Departures from the protocol may happen in any research study and, according to ACCORD item R2, they should be described:

➢ With a list of **each modification** from the plan
➢ The reasons **why** the changes were made

Example 1.

*“Several unique design features of our consensus process included our hybrid approach (eg*, *RAM^25^ and Nominal Group Technique);*^*26*^ *broad guiding questions that required extensive evidence-synthesis; iterative process to develop and revise consensus definitions and recommendations; use of multiple short meetings instead of a traditional singular meeting and videoconferencing*. *Some features were planned ‘a priori’ (hybrid methods*, *broad guiding questions and extensive evidence-synthesis)*, *while others were driven by necessity due to COVID-19 pandemic travel restrictions (multiple short meetings and videoconferencing)*.*”* [49]

Example 2.

*“As mentioned before*, *the definition for an outcome not to be included (termed “consensus out” in Table 1) was changed for the Delphi survey with agreement from the study team*. *There were no other deviations from the protocol*.*”* [53]

Example 3.

***“****As consensus had been reached for all but one item after round 2 and to avoid survey fatigue*,^*16*^ *a third round was deemed unnecessary*.*”* [46]

#### R3. For each step, report quantitative (number of panellists, response rate) and qualitative (relevant socio-demographics) data to describe the participating panellists

The number and characteristics of consensus participants is relevant at all steps of a consensus process as this information serves as a measure of how diverse and representative the participants are of those affected (directly or indirectly) by the consensus recommendations. Participation can change across the various steps involved in consensus exercises making it relevant to report participation details for each step of the process (see Glossary for “Response rate”, “Completion rate”, and “Drop-out”). Note that, in the case of small Delphi panels, care should be taken not to reveal the identities of panellists inadvertently when the number of panellists is low. In cases where panellists could be identified without their prior consent, authors are encouraged to seek further guidance on de-identifying their data.

**Fig 4:**
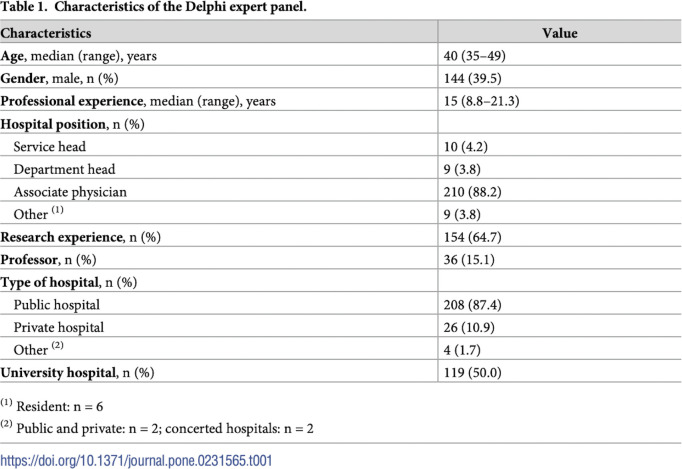
Table by Escobar et al. *PLOS One*. 2020;15:e0231565. (DOI: 10.1371/journal.pone.0231565). This work is licensed under a CC-BY 4.0 licence [80].

**Fig 5:**
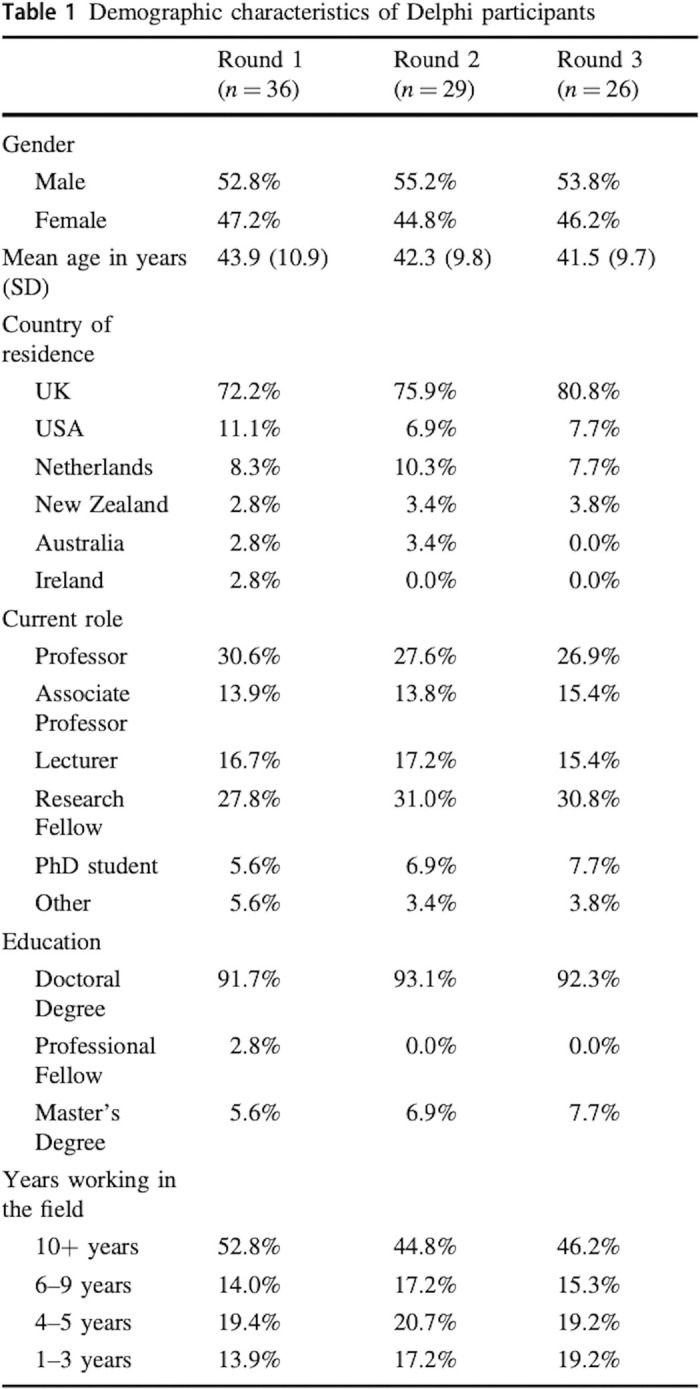
Table by Vogel et al. *Int J Obes*. 2019;43:2573–2586. (DOI: 10.1038/s41366-018-0313-9). This work is licensed under a CC-BY 4.0 licence [42].

In **Example 1**, the authors report the quantitative and qualitative characteristics of the panellists and use a table to assist with concision using text and a table (see **Fig 4**). They do not, however, stratify the data by round, which means that any changes in the overall characteristics of the participant group across the consensus rounds is not apparent. In contrast, **Example 2**, the authors report the number and overall characteristics of the Delphi participants by round (in text and table, see **Fig 5**). To meet the requirements of item R3, it is valuable to report the following information for each step of the consensus process:

➢ The **number** of participants/panellists **invited** to participate (round 1) or who could have taken part (subsequent rounds)
➢ The **number who took part** (the response rate)
➢ The **diversity of the panellists** with respect to the characteristics that made them eligible candidates. Relevant characteristics to consider reporting include panellists’ age, sex/gender, country or region of origin/residence, professional background or experience, and years living with or treating a specific illness or injury.

Example 1.

*“Panel experts*

*A total of 238 cardiologists from 172 hospitals distributed throughout Spain agreed to participate in the project as Delphi panel experts. All 238 experts participated in round 1. Of these, 217 experts completed the round 2 survey. The characteristics of the Delphi expert panellists are summarised in Table 1. Briefly, most participant experts were associate physicians (88.2%) in public hospitals (87.4%) with a median of 15 years of professional experience. Approximately 60% of the cardiologists were involved in research.”*

Example 2.

*“Of the 96 experts invited to participate in this Delphi study*, *36 participants completed Round 1 (37*.*5% response rate)*, *29 of 36 completed Round 2 (80*.*6% response rate) and 26 of 29 completed Round 3 (89*.*7% response rate)*. *Table 1 presents the demographic characteristics of participants in each round*. *Gender distribution was consistent across the three rounds*, *with only a slightly higher percentage of males*. *Participants’ mean age ranged from 42 to 44 years across the three rounds*, *and approximately three quarters resided in the UK*. *The majority of respondents were senior academics*, *had doctoral degrees and had been working in the field of obesity research for ≥ 5 years*.”

#### R4. Report the final outcome of the consensus process as qualitative (for example, aggregated themes from comments) and/or quantitative (for example, summary statistics, score means, medians and/or ranges) data

Consensus exercises are lengthy and often iterative, which means that it is critical to distil the final recommendations clearly so that they can be readily accessed and understood by readers. (See Glossary for “Accessibility”.) The objective of the consensus exercise and consensus method used should guide the final results format, whether quantitative (such as reporting the percentage agreement across specific numbers of statements that panellists voted on), or qualitative (such as narrative summaries of clinical approaches that met the threshold for consensus).

**Fig 6:**
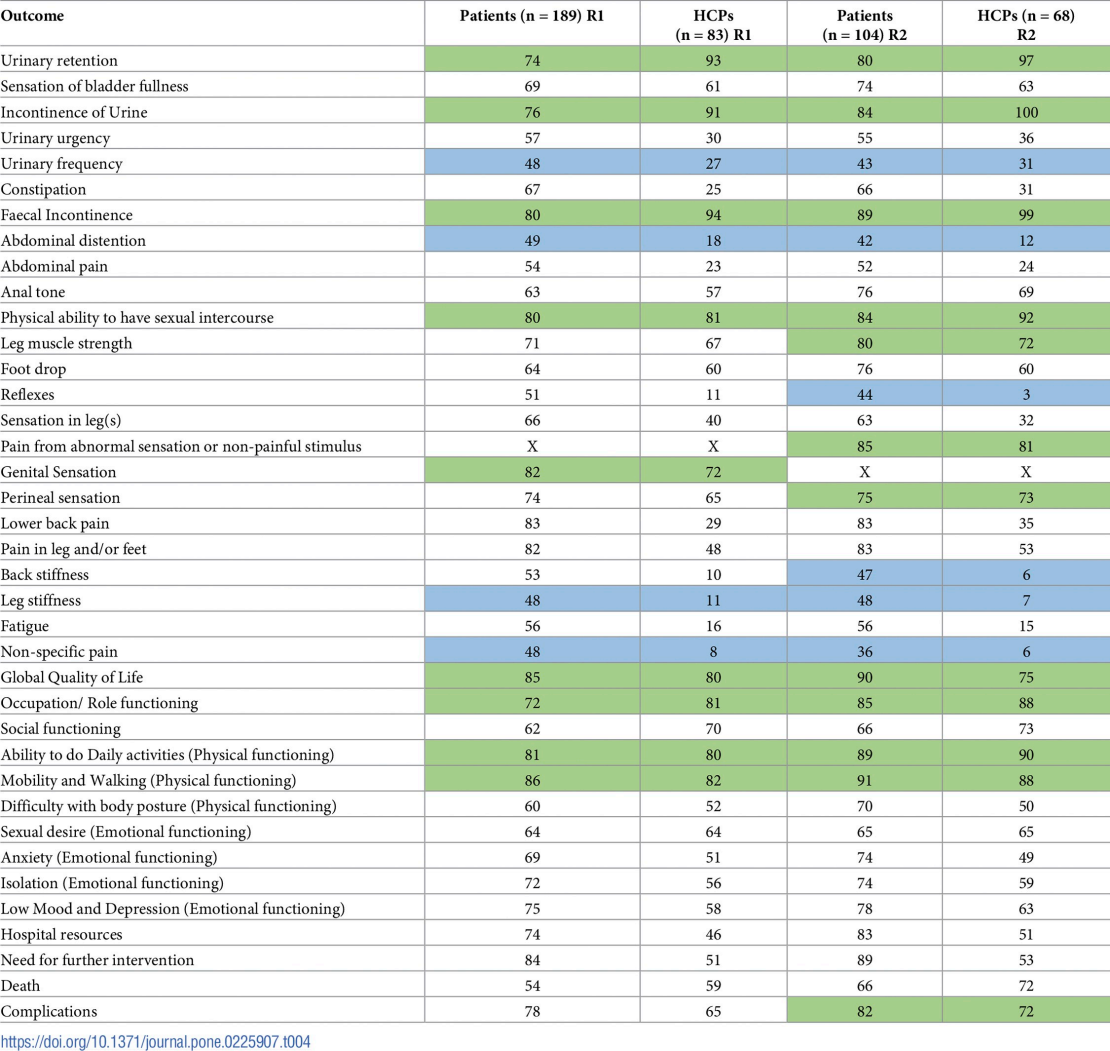
Table by Srikandarajah et al. *PLOS One*. 2020;15: e0225907 (DOI: 10.1371/journal.pone.0225907). This work is licensed under a CC-BY 4.0 licence [53].

**Fig 7:**
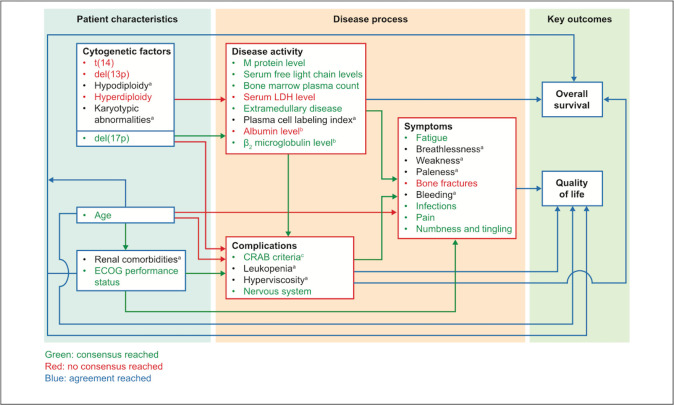
Figure by Gonzalez-McQuire et al. *MDM Policy Pract*. 2019;4: 2381468318814253 (DOI: 10.1177/2381468318814253). This work is licensed under a CC-BY 4.0 licence [81].

**Fig 8:**
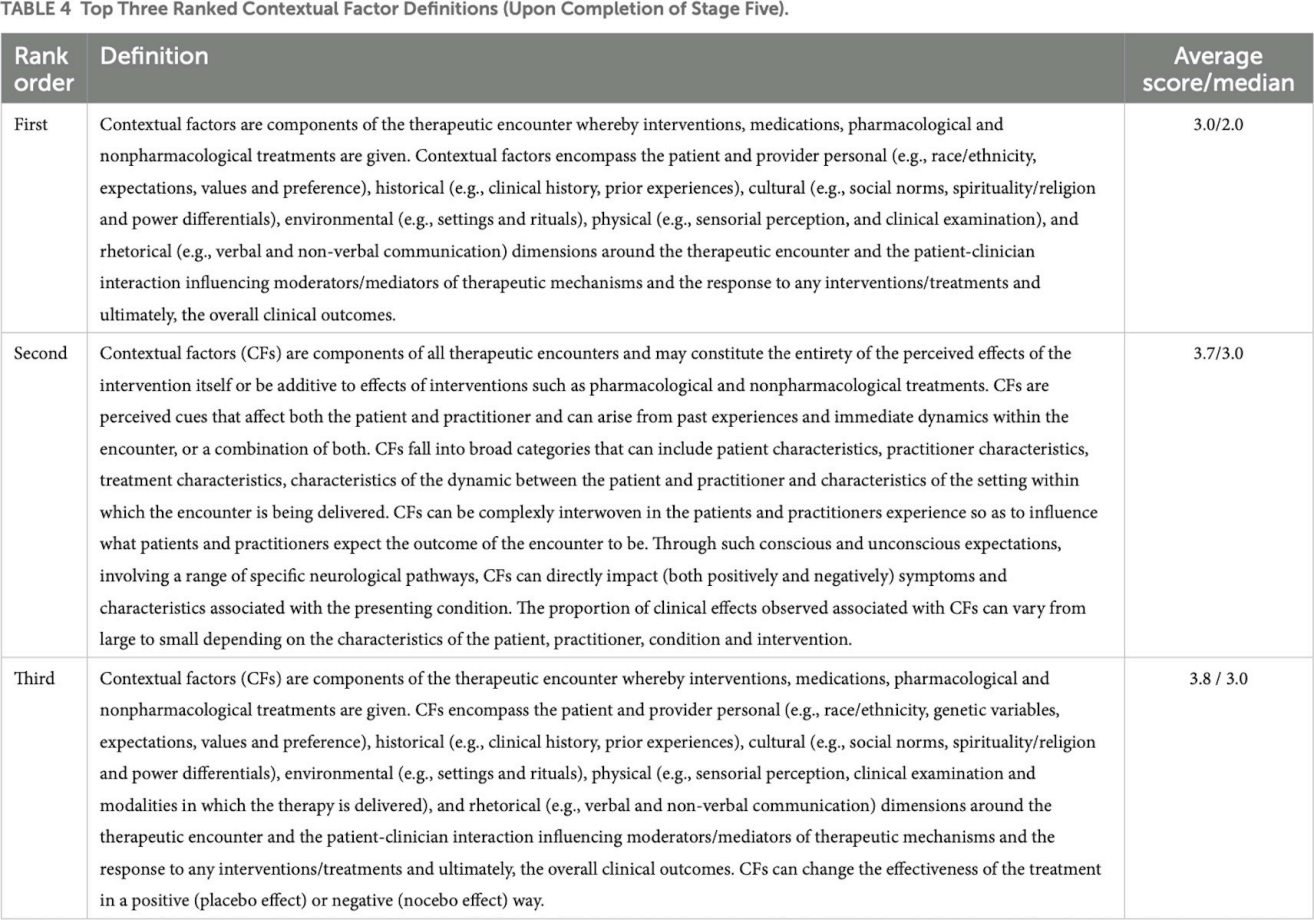
Table by Cook et al. *Front*. *Psychol*. 2023;14:1178560 (DOI: 10.3389/fpsyg.2023.1178560). This work is licensed under a CC-BY 4.0 licence [32].

**Fig 9:**
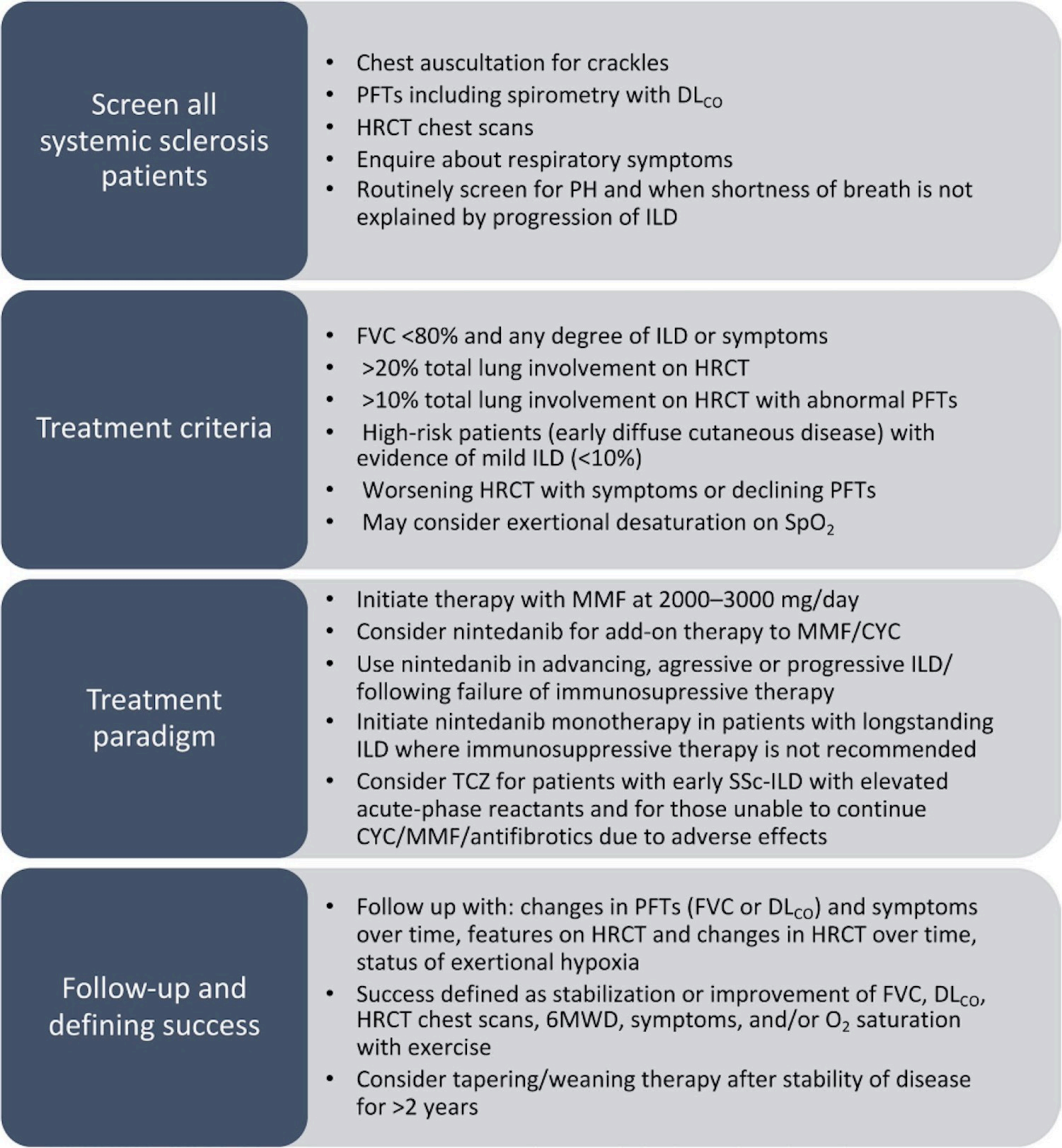
Figure by Rahaghi et al. *Respir Res*. 2023;24:6 (DOI: 10.1186/s12931-022-02292-3). This work is licensed under a CC-BY 4.0 licence [70].

**Fig 10:**
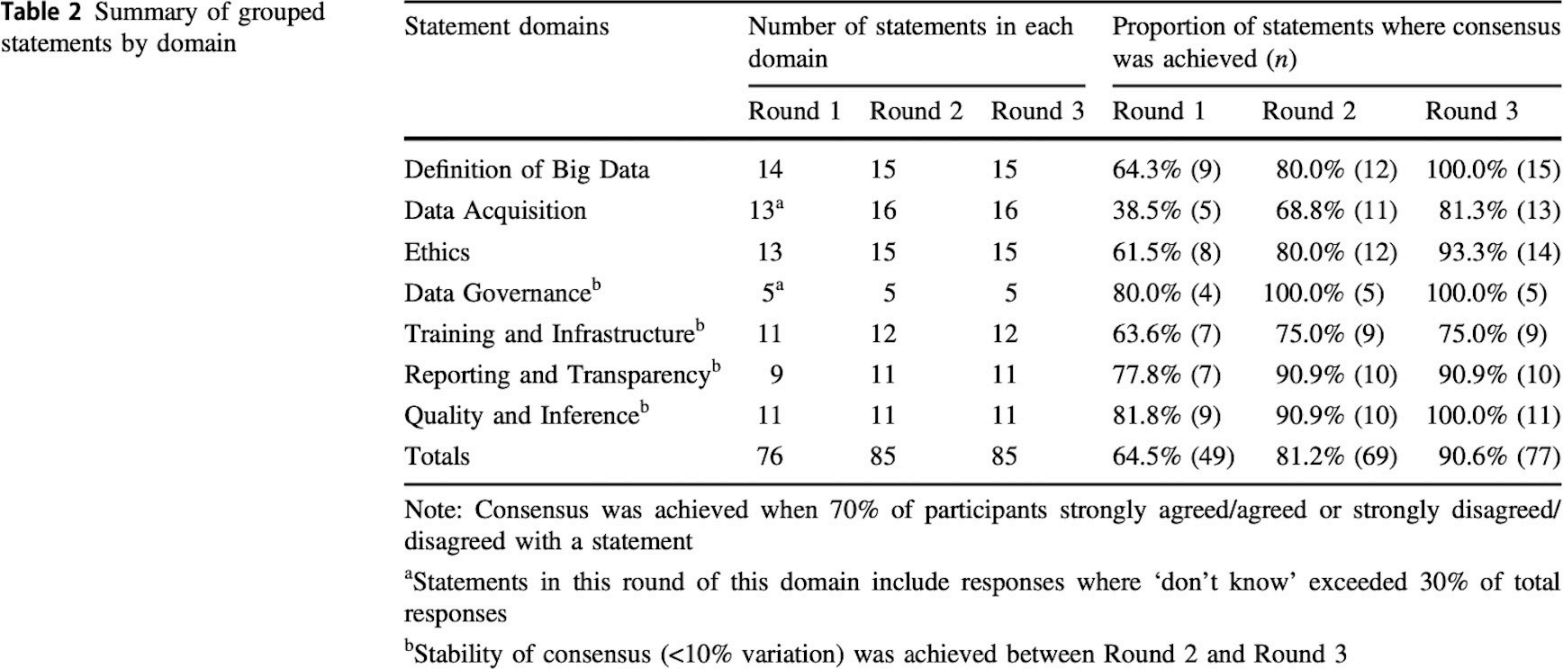
*Table by Vogel et al. *Int J Obes*. 2019;43:2573–2586. (DOI: 10.1038/s41366-018-0313-9)*. *This work is licensed under a CC-BY 4.0 licence [42]*.

In **Example 1**, the authors tabulate all consensus agreements across two Delphi rounds as percentages and indicate, using colour, the items that achieved their definition of “consensus in” (see **Fig 6**). The goal of the authors in **Example 2** was to develop a conceptual model of multiple myeloma, the authors verbally describe factors that were voted in or out before attempting to link them in a map of associations (see text and **Fig 7**)**. Example 3** reports the outcome of the consensus within the text, grouping panellists’ perceptions thematically. **Example 4** illustrates (see **Fig 8**) how quantitative outputs, in this instance form a nominal group technique, can be summarised within the text, complemented by a supporting table. **Example 5** uses the consensus agreements to produce a summary of treatment algorithm (shown in **Fig 9**). Finally, **Example 6** reports the quantitative output from a consensus exercise in a format that demonstrates how the consensus evolved across rounds for each statement domain (see text and **Fig 10**).

In order to fulfil the reporting requirements of item R4, we recommend that authors provide a summary of the consensus findings including either:

➢ **Quantitative summaries**, such as data of percentage agreement across items and consensus rounds; **or**,
➢ **Qualitative summaries**, such as a narrative synthesis produced via thematic analysis
**and**
➢ If appropriate their **recommendations** (including, again if appropriate, the strength of their recommendations)

Example 1.

“*Table 4 shows the percentage of participants who had voted 7 to 9 (critically important) for each outcome at the end of rounds 1 and 2 of the Delphi survey … 13 outcomes were included as “consensus in*,*” (green)*, *6 were “consensus out” (blue) and 19 had “no consensus” at the end of both rounds …*”

Example 2.

*“Consensus was not reached on the direct effect of cytogenetic factors on the disease process; in particular, t(4; 14) and del(17p) were considered to be important prognostic indicators but there was no consensus on their impact on disease activity… Age and comorbidities are heavily interlinked but have been separated in the model because the panelists did not agree about the relationship between age and complications and symptoms: some experts suggested a direct relationship, whereas others suggested an indirect relationship via comorbidities. In the physician-validated model, no consensus was reached on the relationship between age and symptoms and complications; consensus was reached on the impact of ECOG performance status and renal comorbidities on complications and symptoms (Figure 5).*

*… Agreement was reached on the association between age and QoL. The expert who did not agree that age affected QoL commented that elderly patients were more likely to have comorbidities than younger patients, which would reduce QoL*.

*The disease process was separated into disease activity, and complications and symptoms. There was consensus for the factors included in each of these groups and the relationships between them: disease activity affected complications and symptoms; complications affected symptoms. Consensus was also reached that disease activity, comorbidities, and complications affect OS, and that age, comorbidities, and complications and symptoms affect QoL. The physician-validated conceptual model is shown in Figure 5* [here as Fig 7] [81].”

Example 3.

*“From the data collected from the interview, four emergent themes arose regarding good palliative nursing care: (1) the patient and family as a whole, (2) finding meaning, (3) responsible communication and (4) caring for the human element (Figure 2).*

*6.2.1 | (1) Patient and family as a whole*

*In order to approach the care process, the patient and their family should be considered as a whole, which involves, among other things, in-depth understanding of the person in order to be able to help them in their wishes. It also implies availability and collaboration of the different palliative care professionals, to whom this knowledge can be given as a requirement to provide the patient and family with comprehensive and personalised care:*

*‘The first thing in knowing how to care is getting to know the patient and the family very well, that they allow you into the most intimate aspects of their lives, respecting their silence, their time and their wishes. It’s simple. You will get to know a person and they you … your openness’ (I5)*

*‘Family members are an active part in planning care. You enter their home. The family member is present throughout, and we need to remind them that they’ve been there every day, their sensitivity, especially at the time of death, when they fall apart’ (I3)*

*Care units should be generated that empower and work on both; it is as important to work on patients as on their families:*

*‘Patients and family members are constant elements and active agents of continuity of care’ (I2)”* [82]

Example 4.

*“At the end of Stage four (consolidation of ideas), there were 12 definitions that were rank ordered (Table 3). Three definitions were clearly ranked higher than (Table 4) the remaining nine with the majority (80%) of the vNGT selecting these choices as one of the top three selections. These three were similar in content and scope and finished with mean “ranked” scores of 3.0, 3.7 and 3.8, respectively. Following a further poll of the group it was felt that it was necessary to vote again (Round six), but to only include the three definitions. Upon re-vote, one clear winner was identified.*

*Table 4. Top Three Ranked Contextual Factor Definitions (Upon Completion of Stage Five).”*

Example 5.

*“This Delphi study was initiated to develop consensus recommendations for screening, treatment criteria, and the potential role of antifibrotic drugs in patients with SSc-ILD [Systemic sclerosis – Interstitial Lung Disease], building on the latest EULAR scleroderma treatment guidelines and the European consensus statement^12, 14^. The relatively low percentage of statements reaching consensus is reflective of the uncertainty amongst physicians on the appropriate management of SSc-ILD. Nevertheless, the findings from this study provide an algorithm to support effective management of ILD in patients with SSc (Fig 7* [shown here as Fig 9]*), currently the leading cause of death in this population.^7,8^”*

Example 6.

*“Table 2 shows a summary of the Delphi statements for each of the seven domains*. *The number of statements where consensus was achieved improved for each domain from Round 1 to Round 3*. *In Round 1*, *consensus was achieved for 64*.*5% (n = 49) of the 76 statements*. *In Round 2*, *consensus was achieved for 81*.*2% (n = 69) of the 85 statements and this rose to 90*.*6% (n = 77) in Round 3*. *There was variation in the proportion of statements that achieved consensus between domains but the proportion of consensus increased in each subsequent round across all domains*. *By Round 3*, *100% consensus was achieved for three domains (Definition of Big Data (n = 15)*, *Data Governance (n = 5)*, *and Quality and Inference (n = 11); the lowest level of consensus was 75*.*0% for Training and Infrastructure (n = 9)*. *Stability of consensus (<10% variation) was achieved between Round 2 and Round 3 for four of the seven domains*.”

#### R5. List any items or topics that were modified or removed during the consensus process. Include why and when in the process they were modified or removed

During consensus exercises it is expected that statements are retained, excluded, or changed according to the panel’s decisions. A voting round or discussion among participants may prompt the consensus organisers to modify the contents or rephrase their statements to reflect what was approved or disapproved and how (see Glossary for “Disagreement/dissensus”). Reporting on how and in what situations this was done allows the reader to trust that the panel was really listened to. However, it may be easier to report on things that were added or modified for clarity than items that were removed — examples of exclusions are scarce in the literature. ACCORD item R5 asks authors to list both the items removed and modified, and to explain why and when.

**Fig 11:**
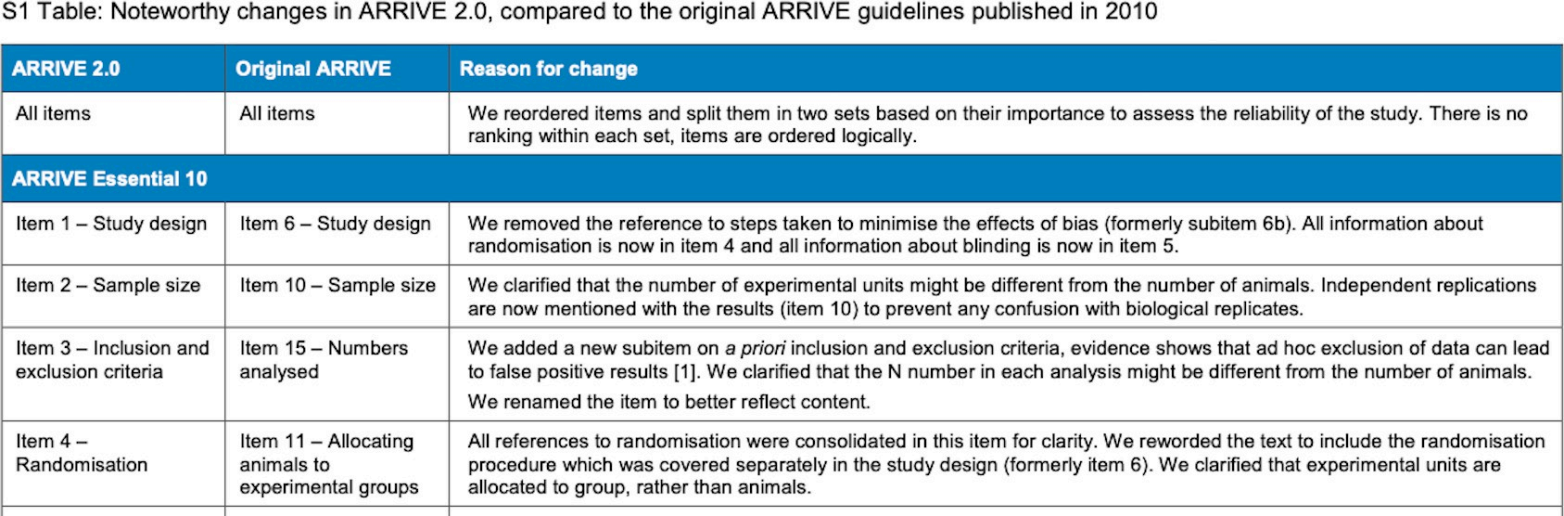
Table by Percie du Sert et al. *PLOS Biol* 2020;18:e3000410. (DOI: 10.1371/journal.pbio.3000410). This work is licensed under a CC0 licence [83].

**Example 1** below is a summary of this type of change reported in a supplementary table (reproduced partially in **Fig 11**). It shows what was changed and explains why, and it mentions removed content. However, it does not give information on when each modification was applied, and it is unclear whether they were all made in the end of the process or as a result of each step [83]**. Example 2**, on the other hand, does explain that the authors had to rephrase the items suggested by the panel after the first panel. This was because the panellists had suggested topics for research that were not formulated as research questions. The statements were then rephrased into questions before the second round of voting. The authors give the full list of topics excluded and rephrased in a supplementary table [84]. When the list of items removed or modified is not extensive, it can be added to the main text, though, as done in **Example 3**, explaining what, why and when [85].

The R5 item of ACCORD checklist reminds authors to be clear about the modifications of the topics throughout the consensus exercise by giving:

➢ **Which** statements/items were changed or removed
➢ **Why** they were changed
➢ **When** they were changed

Example 1.

Example 2.

*“Survey 1 questions were grouped by topic area and rephrased to form answerable research questions (**online supplemental tables S2 and S3**)*. *[…] Questions organised into groups were then rephrased as research questions in collaboration with our public co-authors who ensured that the groupings and rephrasing retained the intent of the original questions*, *and that they were understandable to a lay audience while making them tractable to empirical research*. *For example*, *a question such as ‘Are there medications to treat obesity*?*’ would have been combined with others to become a tractable research question such as ‘What is the effectiveness*, *safety*, *tolerability and cost-effectiveness of medications to treat obesity*?*’”* [84]

Example 3.

*“Ultimately*, *four indicative questions were excluded due to being answered by past research*. *The questions pertained to (1) what interventions are most effective for reducing post-traumatic symptoms among survivors of sexual violence/abuse*, *(2) the relationship between experiencing sexual violence/abuse and having addiction issues*, *(3) whether exposure to sexual violence/abuse leads to short-term and/or long-term mental health problems other than PTSD*, *and (4) the relationship between experiencing sexual violence/abuse and having eating disorders and/or obesity*. *[…] Before rankings were finalised*, *a vote was conducted to merge two thematically related questions concerning how physical healthcare and mental health services could become more ‘trauma informed’ (see questions ranked as ‘7’ in* *online supplemental material 3**)*.*”* [85]

### Manuscript section: Discussion

#### D1. Discuss the methodological strengths and limitations of the consensus exercise

*Include factors that may have impacted the decisions (for example response rates, representativeness of the panel, potential for feedback during consensus to bias responses and potential impact of any non-anonymized interactions)*.

In accordance with other reporting guidelines, authors are requested to consider the strengths and limitations of their consensus exercise. It is important to differentiate strengths and limitations of a methodological approach (for example, ‘the Delphi method allows for iterative development of agreement’) from those of the consensus exercise being reported (for example, ‘the Delphi had a diverse sample of panellists’). Authors should focus on providing the key strengths and limitations inherent in the way *they have applied the methods* within their study which in turn encourages the use of the active voice (I/we).

Strengths may include the inclusion of a very large and diverse sample of panellists, or an expert group of panellists possessing extensive knowledge and authority on the topic. **Example 1** for instance, speaks to strengths arising from a diverse set of panellists but also highlights strengths arising from their process being representative of key groups who can support the implementation of their recommendations.

Limitations might include a failure to employ a planned anonymous voting round leading to the potential for power differentials to effect voting and bias results, a failure to include a key target group who could have been represented in the process or the lack of generalisability of the agreed recommendations (see Glossary for “Generalisability” and “Representativeness”). Authors may also choose to address potential barriers to the implementation of their recommendations as a limitation. **Example 2**, acknowledges problems within the specific context of their own study before recognizing potential limitations associated with the applicability of the study considering they missed their diversity targets during recruitment (see Glossary for “Recruitment”). **Example 3** records where the limitations of the group’s diversity were considered, and how a lack of diversity may influence the transferability of their recommendations.

To report strengths and limitations we recommend that authors:

➢ Provide a balanced discussion of **strengths and limitations specific to the described consensus exercise**, rather than weaknesses of the selected consensus method
➢ Strengths and limitations can also include reflections of the authors on their own involvement and how their attitudes and beliefs may have influenced the consensus development process

Example 1.

*“A strength of our work is that we engaged a diverse group of panelists including academics from disparate disciplines*, *representatives of patient-advocacy organizations and patients*. *The broad endorsement of this statement and pledge by a diverse group of organizations*, *including scientific societies*, *patient-advocacy groups*, *academic and medical centers*, *scientific journals*, *and a parliamentary group provides an unprecedented opportunity for a concerted effort of all stakeholders to effectively tackle this important problem for medicine and society*.*”* [86]

Example 2.

*“While the structured*, *anonymous*, *and democratic approach of the Delphi process offers many advantages to reaching consensus*, *it is not without limitations*. *The methods used here may have impacted our outcome*. *For example*, *the use of a forced choice item rather than a scale in rounds 2 and 3 may have contributed to a greater likelihood for items to reach consensus in these rounds*. *While we endeavoured to attract a diverse and representative sample of institutions to contribute*, *ultimately given our sampling approach*, *it is likely that the participants and institutions that agreed to take part may not be as representative of the global biomedical research culture as we desired*, *and may have a stronger interest in or commitment to open science than is typical*. *While the sample may not be generalizable*, *these institutions likely represent early adopters or willing leaders in open science*. *Further*, *our Delphi surveys and consensus meetings were conducted in English only*, *and the meeting was not conducive for attendance across all time zones*. *These factors will have created barriers to participation for some institutions or participants*. *Defining who is an “expert” to provide their views in any Delphi exercise provides an inherent challenge*.^*21*^ *We faced this challenge here*, *especially considering the diversity of open science practices and the nuances of applying these practices in distinct biomedical subdisciplines*. *For example*, *our vision to create a single biomedical dashboard to deploy at the institutional level may mean we have missed nuances in open science practices in preclinical as compared to clinical research*.*”* [65]

Example 3.

*“Despite deliberate efforts to generate diversity within the expert panel (ie*, *gender*, *race*, *geography and career stage) we acknowledge that we lack perspectives of persons from [non-white] racial groups and from middle to low-income countries*. *Considered alongside the fact that most of the primary studies included in the systematic reviews were conducted in high-income countries*, *the recommendations may have limited applicability beyond white communities and middle- to low-income countries*. *Whenever possible the recommendations include freely available resources (i*.*e*., *PROs [patient reported outcome measures]) and less resource intensive options (i*.*e*., *strength and functional performance testing)*. *The perspectives of patients*, *physiotherapy clinicians and non-physiotherapy clinicians were included from the initial priority setting exercise*, *the evidence synthesis and consensus—however*, *the dominant perspectives represent clinician scientist physiotherapists*.*”* [49]

#### D2. Discuss whether the recommendations are consistent with any pre-existing literature and, if not, propose reasons why this process may have arrived at alternative conclusions

Consensus recommendations should be grounded in the context of any existing research literature. This will help readers to see where they are supported by any research evidence and, importantly, where the group has made contributions to address gaps in the existing evidence. Item D2 may link back to the justification provided in I1 (which asks authors to report why a consensus approach was chosen). It is important to highlight explicitly where opinions diverge from any existing literature (including previous consensus-based recommendations) and why this may have occurred. **Example 1** provides an explicit discussion of where the authors’ updated guideline diverges from previous recommendations and justifies conclusions. **Example 2** grounds the recommendations in the existing literature and clearly demonstrates where there is synergy.

If no research evidence exists this should be recorded, and a broader experiential-based discussion on how the consensus recommendations may impact future clinical and/or research practice may take its place.

Example 1.

*“Our guidelines diverge from the previous OARSI guidelines in 2010 and 2008 as well as from recent American College of Rheumatology (ACR) and European League Against Rheumatism (EULAR) guidelines by focusing specifically on treatment of OA of the knee. The decision was made to examine knee OA separately due to disparities in available evidence between hip OA and knee OA and differences in best treatment practices between these conditions …*

*While many of the recommendations in this guidelines statement agree with those published in other OA guidelines*, *our recommendations differ notably from others in a number of ways*. *Although our recommendations are based on best-available evidence*, *the current evidence contains some areas of inconsistency*. *With regard to non-pharmaceutical treatments*, *our recommendations were largely similar to other recent guidelines published by the American Academy of Orthopaedic Surgeons (AAOS)*, *ACR*, *and EULAR*, *consistently recommending exercise programs for individuals with knee OA as well as weight loss programs for overweight individuals with knee OA*. *For this guidelines statement*, *exercise modalities were divided into three groups (land-based*, *water-based*, *and strength training) to provide greater specificity than other OA guidelines in assessing their distinct benefits and risks and to evaluate their relative appropriateness for different clinical sub-phenotypes*. *In other areas of non-pharmacological treatment*, *our guidelines differed more substantially from others*. *For electrotherapeutic modalities*, *AAOS provided an ‘Inconclusive’ recommendation*, *while these guidelines recommend against the use of TENS and provide an ‘Uncertain’ recommendation for EMG biofeedback*. *While ACR conditionally recommends acupuncture for knee OA*, *and AAOS does not recommend acupuncture*, *our guidelines provide an “Uncertain” recommendation regarding acupuncture*, *highlighting the lack of strong available evidence regarding its use*. *Recommendations regarding biomechanical interventions were also mixed; AAOS provided an inconclusive recommendation regarding force braces*, *and both AAOS and EULAR recommended against the use of wedged insoles*, *while ACR conditionally recommended the use of medially wedged insoles*. *Rather than providing recommendations individually for specific biomechanical modalities*, *these guidelines recommend the use of biomechanical interventions as directed by an appropriate specialist*.*”* [36]

Example 2.

*“The WHO definition of post-COVID-19 condition*^*3*^ *includes the most prevalent symptoms*, *such as fatigue*, *shortness of breath*, *and cognitive dysfunction*, *which generally have an effect on everyday functioning*. *Fluctuating or relapsing symptoms are also commonly reported*.^*22*^ *As reflected in the WHO definition*, *people with post-COVID-19 condition can have many other symptoms*. *Eight of the eleven consensus-based outcomes in the COS [core outcome set] presented here are in the physiological or clinical outcomes domain and cover all of the most prevalent symptoms reported in existing research*. *This COS complements the WHO definition because both are aiming for harmonisation of clinical research and practice for long COVID*. *The WHO definition provides a standardised term for post-COVID-19 condition*, *while the COS identifies the minimum outcomes that should be measured in all research studies and clinical practice*.*”* [40]

### Manuscript section: Other information

#### O1. List any endorsing organisations involved and their role

Endorsement by relevant organisations is often sought when developing clinical guidelines (see Glossary for “Clinical practice guideline”). Endorsement by a reputable organisation (frequently a medical society or similar group) is often a ‘stamp of approval’ and can support the adoption of recommendations. However, endorsement processes vary, and as such, organisational approval should not automatically be interpreted as an assurance of rigour and quality. Moreover, organisations are not always impartial in the development of recommendations and guidelines. Therefore, their explicit level of involvement should be documented.

To ensure transparency, all sources of non-financial support and endorsing organisations involved in the consensus exercise should be reported along with their role. (See Glossary for “Advisory board”, “Executive committee” and “Steering committee”.) This endorsement is different from financial support or funding, which should be reported separately (as per item O3 below). ACCORD item O1 requests information on general support of the initiative and endorsement of the resulting recommendations.

In the following examples, **Example 1** includes a detailed description of all organisations and persons involved in the development of the guideline and the steps involved in securing endorsement of the work. **Example 2** simply states the name of the organisation that endorsed the recommendations and that the endorsement was informed by the opinion of specialist reviewers.

Example 1.

*“This clinical practice guideline on the management of BAD [bile acid diarrhea] was developed under the direction of Dr Daniel Sadowski*, *in accordance with the policies and procedures of the Canadian Association of Gastroenterology and under the direction of the Canadian Association of Gastroenterology Clinical Affairs*. *It has been reviewed by the Canadian Association of Gastroenterology Practice Affairs and Clinical Affairs Committees and the Canadian Association of Gastroenterology Board of Directors[*…*] As per Canadian Association of Gastroenterology policy for all clinical practice guidelines*, *the manuscript was made available to all Canadian Association of Gastroenterology members for commenting before submission for publication*. *Members were notified that the manuscript was available on the members-only section of the Canadian Association of Gastroenterology website and open for comment for a 2-week period*.*”* [57]

Example 2.

*“These recommendations are endorsed by the National Cancer Research Institute*, *and have been reviewed by oncologists from regional cancer centres across the United Kingdom*.*”* [87]

#### O2. State any potential conflicts of interests, including among those directing the consensus study, and panellists. Describe how conflicts of interest were managed

Conflicts of interest have the potential to negatively affect the integrity and credibility of the consensus exercise (see Glossary for “Conflict of interest”). Therefore, information on all potential conflicts of interest should be disclosed for all those involved in the consensus, including panellists and those directing the consensus exercise. Potential competing interests include financial conflicts, non-financial conflicts and conflicts of role which can be perceived to have an influence on the actions, judgment and/or decision-making due to vested interests. A description of how competing interests were managed should also be reported as it provides transparency as to whether any measures were implemented to try to minimize the potential impact of real or perceived conflicts.

For those involved in leading the consensus exercise, the potential impact of competing interests may be diluted by ensuring that decisions are made by a leadership group or subgroup rather than by a single individual. Where conflicts of interest might be thought to influence the judgement of panellists on some voting topics, the panellist might be, for example, excluded or they may recuse themselves from voting on related items.

In **Example 1**, the authors report what types of conflicts of interest were disclosed by participants, when during the consensus steps these were captured, and updated, and also how they were assessed and managed. **Example 2** states the approach/policy used to define conflicts of interest, describes how potential conflicts of interest were assessed for members of the guideline development group, and how relevant conflicts of interest were managed. As for Example 2, **Example 3** refers readers to an external policy/convention that was used to define what constituted a potential conflict of interest. The percentage of participants who declared conflicts of interest in accordance with the stated classification system is reported, as are the resulting limitations that were placed on those individuals’ participation during in the consensus process.

To meet the reporting requirements of item O2, we recommend that authors:

➢ **List all conflicts** of interests for individuals involved in the consensus exercise, specifying their role in the work
➢ State whether any measures were taken to **mitigate** against real or perceived conflicts of interest.

Example 1.

*“Management of conflict of interest (COI)*

*At the request of the OARSI [Osteoarthritis Research Society International] Ethics Committee*, *all members of the OAGDG [Osteoarthritis Guidelines Development Group] were required to complete a COI questionnaire to report any potential conflicts including consulting*, *grant support*, *practice revenue*, *intellectual property*, *etc*. *for each treatment (Appendix 1)*. *During initial rounds of voting*, *OAGDG members were instructed to recuse themselves from voting on potentially conflicted treatment modalities*. *At the April 2013 OARSI meeting*, *OAGDG members updated disclosures and discussed these conflicts in person with an ethics committee member prior to the final round of voting*. *The Ethics Committee representative made a final determination regarding the level at which a potential conflict would disqualify an OAGDG member from voting on each treatment*. *Final disclosure and voting recusal results were twice distributed among the OAGDG to verify their accuracy*.*”* [36]

Example 2.

*“The CTS [Technical Scientific Committee] applied the COI policy included in the CNEC [National Centre for Clinical Excellence, Quality and Safety of Care] methodology, which is consistent with international recommendations.^12,19,23^ The COI disclosure was used to assess the eligibility of each member of the GDG [guideline development group] by the CTS and it was PICO specific for Panel members. Moreover, the COI declared by each panellist was classified in 3 degrees of relevance. COIs classified as of “minimal or insignificant relevance” posed no limitation to participation in all phases of the recommendation development process*.

*Panellists whose COI was classified as “potentially relevant” were admitted to all the process phases but required a public disclosure in the final document or in the SNLG website*.

*Finally, COIs classified as “relevant” led to a partial exclusion from the participation to discussion and voting of the criteria potentially influenced by the specific COI, up to the complete exclusion from the Guidelines Development process.^19^*

*Moreover, the use of the GRADE Evidence-to-Decision framework minimized the influence of COI on recommendations’ formulations, leading the experts to make informed choices based on predefined and transparent criteria.^13,19^*

*Finally*, *panellists refusing to fulfil and sign the COI disclosure form or not participating to the plenary session were excluded from the authors’ list of each specific recommendation*.*”* [88]

Example 3.

*“38% of the experts declared personal-financial interests (for details on classification see EuroGuiDerm Methods Manual v1*.*3*.*)*. *These members were neither eligible to take the lead in a respective working group nor for voting on recommendations pertaining to systemic treatment on the stepped-care plan*.*”* [89]

#### O3. State any funding received and the role of the funder.

*Specify, for example, any funder involvement in the study concept/design, participation in the steering committee, conducting the consensus process or funding of any medical writing support. This could be disclosed in the methods or in the relevant transparency section of the manuscript. Where a funder did not play a role in the process or influence the decisions reached, this should be specified*.

Reporting all sources of funding received for a consensus exercise ensures transparency around any potential conflicts of interest (see reporting item O2) and bias that might have influenced the design, conduct and/or findings of the consensus exercise. Sources of funding can be ‘hands off’, such as where a funder provides a grant for the work but has no involvement in the design or delivery of the research. Alternatively, funding may be conditional and/or include both financial or in-kind support and some degree of involvement or approval of the consensus approach. Types of funding that are relevant to disclose include, but are not limited to: research grants; payment of personal fees (consulting fees, for time spent and/or lost income); payments for manuscript writing or translations, article processing charges (open access fees); provision of services (for example, in-kind support, tools, devices for voting, space or software) and payment of travel expenses, out-of-pocket costs, meeting costs.

**Example 1** reports who funded the research and describes the funder’s role at all stages of the process, including the involvement of employees of the funding organisation**. Example 2** also discloses all sources of funding and support received, and reports whether these funders/supporters had any influence on different aspects of the work. Finally, in **Example 3**, the sources of funding for different parts of the project (including the consensus exercise) are reported and the authors make clear that the funder(s) played no role in the conduct of the work.

We suggest that authors specify, for each and all sources of funding received:

➢ The **name** of the funder
➢ The **nature** of the funding provided (what was paid and to whom)
➢ Any **role** that the funder played in the consensus exercise and its reporting

Example 1.

*“The Delphi consensus was coordinated by a healthcare consulting and training company (Sanitanova Srl*, *Milan*, *Italy)*. *The consensus concept was initiated and funded by Merck KGaA*, *Darmstadt*, *Germany*. *The sponsor was involved early in the process*, *defining the overarching topic to be discussed*, *but did not participate in the development of the statements or in any of the meetings or discussions involved in developing the Delphi consensus*. *The statements were*, *therefore*, *developed independently of the industry sponsor*. *The authors from Merck KGaA*, *Darmstadt*, *Germany*, *were only involved in the development of the manuscript*, *critically revising it for important intellectual content*, *especially in the Introduction*, *Results and Discussion sections*, *but could not alter the consensus statements in any way*.*”* [63]

Example 2.

*“This work was supported by the Sanofi-Genzyme and Regeneron Alliance*. *The Sanofi- Genzyme and Regeneron Alliance provided funding for the 3AD program but had no influence on the development of the recommendations*. *Sanofi-Genzyme and Regeneron had no influence on the development of the manuscript nor did they review the content of the manuscript*. *The authors (all of whom are members of the 3AD Steering Committee) determined and approved the final content of the manuscript*. *The Steering Committee has been assisted by Lighthouse Medical Communications US (formerly known as Lucid US)*, *a specialist medical communications company*, *which was funded by Sanofi-Genzyme and Regeneron*, *for the organization of the program and editorial support of the manuscript*. *The authors maintained complete control over the direction and content of the paper*. *No payments were made to the authors for the writing of this manuscript*.*”* [90]

Example 3.

*“Funding The author(s) disclosed receipt of the following financial support for the research*, *authorship*, *and/or publication of this article*: *This research was commissioned by Oxford Health Policy Forum*, *on behalf of the MS Brain Health initiative*. *Grants to support MS Brain Health were provided by Actelion Pharmaceuticals*, *Biogen*, *Celgene*, *F*. *Hoffmann-La Roche*, *Merck KGaA and Sanofi Genzyme*, *all of whom had no role in study design*, *data collection*, *data analysis*, *data interpretation*, *writing of the report or in the decision to submit the paper for publication*.*”* [91]

**Box 1: Glossary of terms defined in the context of consensus exercises**

The definitions and descriptions given in this glossary relate to their use in the specific context of the ACCORD reporting guideline. They are not necessarily applicable or to other areas of research or education.

**Accessibility**: in consensus research, the concept of accessibility can relate to three areas: the process; the materials developed to facilitate the process; or the published results. Accessibility to the consensus process refers to the ability to participate in the different parts of the exercise (physically and/or virtually), and whether all groups affected by the consensus were facilitated to participate. In this context, accessibility can be improved when participants are offered to have their transportation costs covered, or access to the internet or online platforms, for example. Accessibility of the materials developed to facilitate the exercise refers specifically to the extent to which the information used is understandable and written in unbiased language that is free of expert or technical jargon [92]. To improve accessibility of materials, project leaders can for instance: develop materials in different languages or dialects; use different formats (graphics, video, audio); or provide additional explanations or help for the participant to interact. Accessibility of the results refers to whether the authors publish final results in open access journals or another format that is free for the public to read.

**Advisory board:** an external group of people who act as consultants, offering advice based on their expertise (skills and knowledge) and/or experience (prior involvement) of the consensus approaches and/or the subject of the consensus activity. Most often this group reports directly to the steering or executive committee. See also ‘Executive committee’ and ‘Steering committee’.

**Anonymity:** ensuring that participants’ identities remain unknown or disconnected to their votes. The purpose of preserving anonymity is to reduce the potential for dominant or authoritative personalities to bias or lead a group to a particular compromise [93], vote or conclusion. Within consensus research, anonymity is a core pillar of the Delphi process, but may also be present in other methods. Anonymity may apply to panel participants (blinding them to each one’s identity and/or votes) and/or to the researchers conducting the consensus (blinding the researchers to the identities of the participants).

**Consensus threshold:** the value signifying that agreement (consensus) has been reached among the group of panellists or stakeholders. A threshold value is an amount, rate, level, or limit on a scale decided by the consensus organisers [94].

**Clinical practice guideline (CPG):** document that provides recommendations for optimizing patient care. CPGs comprise systematically developed statements or processes to assist clinician and patient decisions about what constitutes an evidence-based approach to healthcare. CPGs should be developed through a rigorous procedure of audit, literature search and grading of the quality of the available evidence (predicated on certainty of the available evidence) to improve the standards of diagnosis, treatment and management of specific diseases and conditions.

**Completion rate**: relates to the proportion of participants who were invited to participate and took part in the complete consensus exercise (one meeting until its end, or all questions in one Delphi round, for example). To be meaningful, the completion rates must be reported for all steps, phases, sessions or rounds; for example, the proportion of participants who complete the first round of voting is likely to differ from the proportion who completed all voting rounds, which may in turn differ from the number who participated in different sessions of an in-person consensus exercise. It should be clear at each stage who was invited (denominator) and who completed (numerator). See also ‘Response rate’.

**Conflict of interest:** exists when a participant involved in a consensus exercise (panel member, facilitator, steering committee member, author) has a relationship/competing interest that may be viewed as influencing (or be reasonably seen to do so) their responsibilities in the unbiased design/conduct/reporting of a consensus exercise, or in voting. Competing interests include (but are not limited to) academic commitments, personal relationships, political or religious beliefs, and institutional affiliations, and financial ties [95].

**Disagreement/dissensus:** non-agreement or opposition to an idea, or principle of action, due to differing views among consensus exercise participants.

**Dominance/peer pressure:** the concept that an individual (or a group of individuals) has the power to influence the opinion or have undue influence over other participants. Factors that can contribute to dominance can be strong verbalization by the dominant individual/subgroup, not allowing time/space for individuals and/or subgroups to express themselves or not allowing them to register their views. They can also be passive differences related to culture, age, and/or professional seniority.

**Drop-out:** an individual who takes part in part of the consensus exercise, but who does not continue to the end of the process. Defining the parameters for drop-out is important. For example, in a consensus exercise comprising multiple voting rounds or meetings, the individual could drop out if they take part in the first, but not subsequent rounds of voting, or they come to the first two meetings and not the last one. Drop out is closely related to the ‘Completion rate’ (see above).

**Element:** The ACCORD guideline comprises 36 reporting items. Each of these items may require the reporting of more than one piece of information; each of these pieces of information are referred to in this document as ‘elements’. For example, ACCORD item I2 asks for three elements: aim, intended audience, and location.

**Executive committee:** A group of people with specific executive or administration roles, for instance to implement actions to make sure the consensus exercise as a whole meets its targets and deadlines in practice and communicates with members of other committees. The executive committee may or may not be part of other groups in the consensus exercise, such as the steering committee or advisory board, and may share responsibilities with them. See also ‘Project committee’ and ‘Steering committee’.

**Expertise/expert:** refers to the depth of knowledge on a topic or concept. An individual with expertise (often validated by an external measure such as a qualification, level or years of professional practice – see next item) has substantial knowledge and/or skills in an area pertinent to the design, conduct and/or subject of the consensus exercise.

**Experience:** speaks directly to the amount of exposure an individual has to the topic as opposed to their knowledge (which is described by ‘expertise’). Most often measured in years of exposure (‘years of experience’). Experience may also be used to define who is an expert, based on their exposure to the topic. It is frequently used to refer to patients with experience in specific conditions (for example, pregnancy), situations of care (use of a hospital’s service) or health problems (a disease or trauma).

**Facilitator/facilitation:** Someone who supports consensus meetings by facilitating the discussion or making sure, in more structured techniques, that relevant steps are followed. The facilitator should remain neutral in the decision-making processes; their role is to guide the consensus group through the exercise, helping them to retain focus and enabling them to perform their role(s) and tasks. A facilitator may be an expert on the topic under consideration, or simply an expert facilitator (someone who has a qualification or demonstrated ability in facilitating groups). See also ‘Mediation/mediator’.

**Fatigue/survey fatigue:** in the context of the development of a consensus statement, survey fatigue is when respondents lose interest in a survey due to being asked to complete an excessively long survey, multiple rounds, or multiple surveys. This type of fatigue can lead to low response rates, rushed completion (particularly of later questions/stages), or abandonment (drop-outs), which can have an impact on survey results [96].

**Generalisability**: sometimes referred to as ‘external validity’ or ‘applicability’, is the extent to which the outcome of a consensus exercise can be applied to other circumstances [97]. The term is used to discuss clearly specified conditions such as the extent to which consensus outcomes developed in one country or population may be directly applied to healthcare systems in other countries, or to a population that differs in some way (for example, for age, sex, ethnicity, clinical status) [18,98].

**Iteration:** is the repetition of a process or steps within the consensus exercise. The repetition is done to improve upon previous versions of a recommendation or group agreement. Iteration is a central tenet of the Delphi method and in that method is represented by the repeated rounds of voting to allow panellists to ‘iterate’ on their previous votes based upon feedback.

**Likert scale:** a psychometric scale frequently used in surveys, including Delphi, to measure the level of agreement with statements, ‘feelings’ or other qualitative phenomena. For example, in response to a statement a person can indicate that they ‘strongly disagree’, ‘somewhat disagree’, ‘are undecided’, ‘somewhat agree’, or ‘strongly agree’ [99]. Likert scales have an odd number of categories such that an equal number of alternatives lies on either side of an intermediate value, always allowing participants to express neutrality.

**Mediator/mediation:** when members of the consensus are in conflict about an issue, mediation is a procedure or situation in which a person external to the conflict, known as the mediator, is called to intervene and help to solve the dispute by finding a conciliating solution for the problem. The mediator has to be a neutral arbitrator who does not take sides or favour any member of the consensus group [100]. The mediator differs from the facilitator, as a facilitator’s goal is to remain neutral and impartial, whereas the mediator will engage both sides to try and resolve disagreement. See also ‘Facilitation/facilitator’.

**Panel:** the group of individuals with relevant expertise or experience who are invited to take part in a consensus exercise by expressing their agreement (or disagreement) with specific topics, decisions or statements and attempt to reach a set of recommendations, for instance, to agree on a set of minimum reporting criteria when studying a disease, or on the best-practice treatment of an injury or illness.

**Project committee**: often subordinate to the steering or executive committee. A small group of people directly involved in one part of the consensus exercise and/or who provide administrative oversight of it (including organisation of meetings, activities, keeping documentation, or sending reminders for project deadlines). The project committee may also be divided into smaller ‘working groups’ who are designated to work on specific tasks that support the overall consensus exercise. See also ‘Executive committee’ and ‘Steering committee’.

**Ranking:** positioning items within a hierarchy or scale. Ranking items requires them to be compared directly and positioned relative to each other. For example, ranking three items requires them to be positioned first, second and third.

**Rating:** provides detail about the quality of items using a common evaluation scale. Rating three items using a scale of 1–10, for example, requires an independent appraisal of each item against the parameters of the scale. Unlike ‘ranking’, rating items may allow for several different items to have the same score. For example, asked to rate statements on their level of importance using a scale of 1-10, panellists may attribute the same score 10 to all three, suggesting all of them have equal and high importance.

**Recruitment:** the strategy, process and method used for identifying, reaching, and effectively inviting people to participate. This should not be confused with ‘including’ individuals in a consensus panel, so the number of participants recruited and the number of individuals who actually participated (were included) frequently differ.

**Reporting guideline:** a document developed to guide authors on the minimum information to include when publishing research. A reporting guideline specifies, as a checklist, flow diagram or structured text, the information that should be shared with readers to allow them to understand how the research was performed, its strengths and limitations, and how to reproduce the research. ACCORD is a reporting guideline for consensus exercises.

**Representativeness:** the extent to which a sample of a group represents the characteristics of that larger group, or population. In the context of consensus research, the concept refers applies to the extent to which the participants or panellists of the consensus exercise represent the characteristics of the larger population of individuals who will be affected by the consensus results.

**Response rate:** the proportion of individuals who responded to a particular invitation or survey question. There may be cases where someone responds to the first question(s) in a Delphi survey (allowing authors to report the response rate per item) but not all of them in the Delphi round (which reduces completion rate). There may also be situations where individuals respond an e-mail agreeing to an invitation — which shows recruitment efforts reached them — but were, in the end, unable to travel to a meeting, for example — which affects the completion rate. See also ‘Completion rate’.

**Round robin:** most often part of nominal group technique (NGT). The round-robin refers to step two of NGT where the ideas of panellists are collected and recorded one at a time, and in sequence. Each panellist is asked to contribute an idea on the topic of discussion until all members have had an opportunity. The process is repeated until no further novel ideas emerge from the group of panellists [12].

**Sample size**: in the context of consensus, the number of individuals involved in each consensus-reaching step of the exercise. It can be the number of panellists in a Delphi round, in one or a sequence of meetings, or other consensus activities [101].

**Snowball sampling:** an approach that relies on peer referral for recruitment and/or to help reach the target sample size or target demographics/characteristics. In this approach, researchers select initial participants (called seeds) who recruit their peers, who then themselves recruit their peers, and so forth until the target sample size is reached or target demographics are achieved. The approach can be effective at recruiting hard-to-reach groups [102]. In the context of consensus exercises, snowball sampling may be used to help recruit individuals to participate in the consensus steps (for example, panellists in the case of Delphi approaches).

**Stability**: the consistency of responses between successive rounds of a consensus exercise. Responses may be unstable between rounds due to individuals changing their position, even if the overall level of agreement, an aggregate measure for panel as a whole, remains consistent [103].

**Steering committee**: a group who takes responsibility and is accountable for the strategic planning of a consensus study, including its methodology, participants, steps and resources. Members of the steering committee make decisions based on the study objectives, ethics and oversight the actions of executive and project committees. Some of its members can also take executive roles. The steering committee may also request the input from an advisory board. See also: ‘Executive committee’, ‘Project committee’, and ‘Advisory board’.

## Supporting information

S1 Table Problems with word count limits imposed by journals and tips on reporting all reporting guideline checklist items. (DOCX)
