# Supplementary material for: ACcurate COnsensus Reporting Document (ACCORD) explanation and elaboration: Guidance and examples to support reporting consensus methods
Source: PLoS Med. 2024 May 6;21(5):e1004390. doi: 10.1371/journal.pmed.1004390 (PMC11198995; doi:10.1371/journal.pmed.1004390)
Supplement: S1 Table — (DOCX) [file pmed.1004390.s001.docx]

**S1 Table: Problems with word count limits imposed by journals and tips on reporting all reporting guideline checklist items**

| **Problem/issue** | **Argument: the reasons why** | **Solutions** |
| --- | --- | --- |
| **journal article word count limits** | Word count limits imposed by journals should not prevent the reporting of all checklist items in a reporting guideline, including ACCORD. | **Use open research platforms** — Instead of omitting information, especially in the methods and results section, researchers should make sure they report all checklist items as a minimum. The use of all the spaces currently available as research publication options, most of them for free, gives authors a lot of freedom. The recommendations below not only represent open research practices but also help in reducing the total word count while still reporting completely. |
| **Techniques or procedures details: protocols are long** | A detailed description of the study methods overall and each procedure or technique used is crucial for the evaluation of bias and for reproducibility of the study in other settings and populations. | **Publish a protocol** — Publishing a protocol before conducting the consensus exercise or the study is recommended as a registration of a study plan and intents. By making the detailed protocol publicly available, and preferably open access, authors can cross-reference it and give only a brief summary in the ‘results’ paper. Readers who wish or need to know more can easily access the detailed protocol. Changes made to the protocol can and should be noted in the results paper, as recommended by ACCORD. The protocol can be published as a preprint or peer-reviewed journal article, both providing a unique digital object identifier (DOI) for simplified cross-referencing, durable access, and easy citation. |
| **Results tables occupy too much space** | All data from health research, summarised or not, should be available to allow verification and use in clinical practice. | **Use supplementary materials** — Journals impose word count limits to the main research article, but they usually allow the use of supplementary materials. Supplementary materials are easily accessed from the main text and can provide a detailed description of the results or procedures used. These can be tables, lists, qualitative results (as quotations or whole declarations from participants) and other materials. Authors can produce neat formatted supplementary materials for peer review and publication. Although they are not formatted in the journal’s visual style in the final article pdf, and usually not copyedited, they can be linked to the main article and stored in the journal’s servers. Readers can then easily access them clicking on the article links. |
| **Datasets are not published by journals** | Whole datasets or separate raw data tables and images are useful for other researchers conducting reproducibility or new studies. They are also important for statistical peer review. Except for data impossible to anonymise, in health research all datasets should ideally be publicly available immediately (and not only ‘under request’). | **Deposit data** — Several platforms today allow the deposit and sharing of datasets, and many offer each part of it its own DOIs. This means these datasets (such as participants’ characteristics, test results, disaggregated responses, videos, and others) can be easily cross-referenced in the journal article. Citing datasets with their DOI numbers shows transparency in research and helps with journal limits, as they do not count as one table, only as one reference citation. The Open Science Framework (<https://osf.io/>) is just one example of platform that allows the publication of multiple types of data, including text, original data extraction and cleaning files, and images (photo and video), all under one single research project umbrella. In some platforms, authors can set special permissions for restricted access when this is necessary to protect privacy. |
